# Supplementary material for: MYCN amplification and ATRX mutations are incompatible in neuroblastoma
Source: Nat Commun. 2020 Feb 14;11:913. doi: 10.1038/s41467-020-14682-6 (PMC7021759; doi:10.1038/s41467-020-14682-6)
Supplement: Supplementary file 1 — Supplementary Information [file 41467_2020_14682_MOESM1_ESM.pdf]

Supplementary Information

***MYCN* Amplification and *ATRX* Mutations are Incompatible in Neuroblastoma**

Zeineldin, M et al.

SUPPLEMENTARY FIGURES

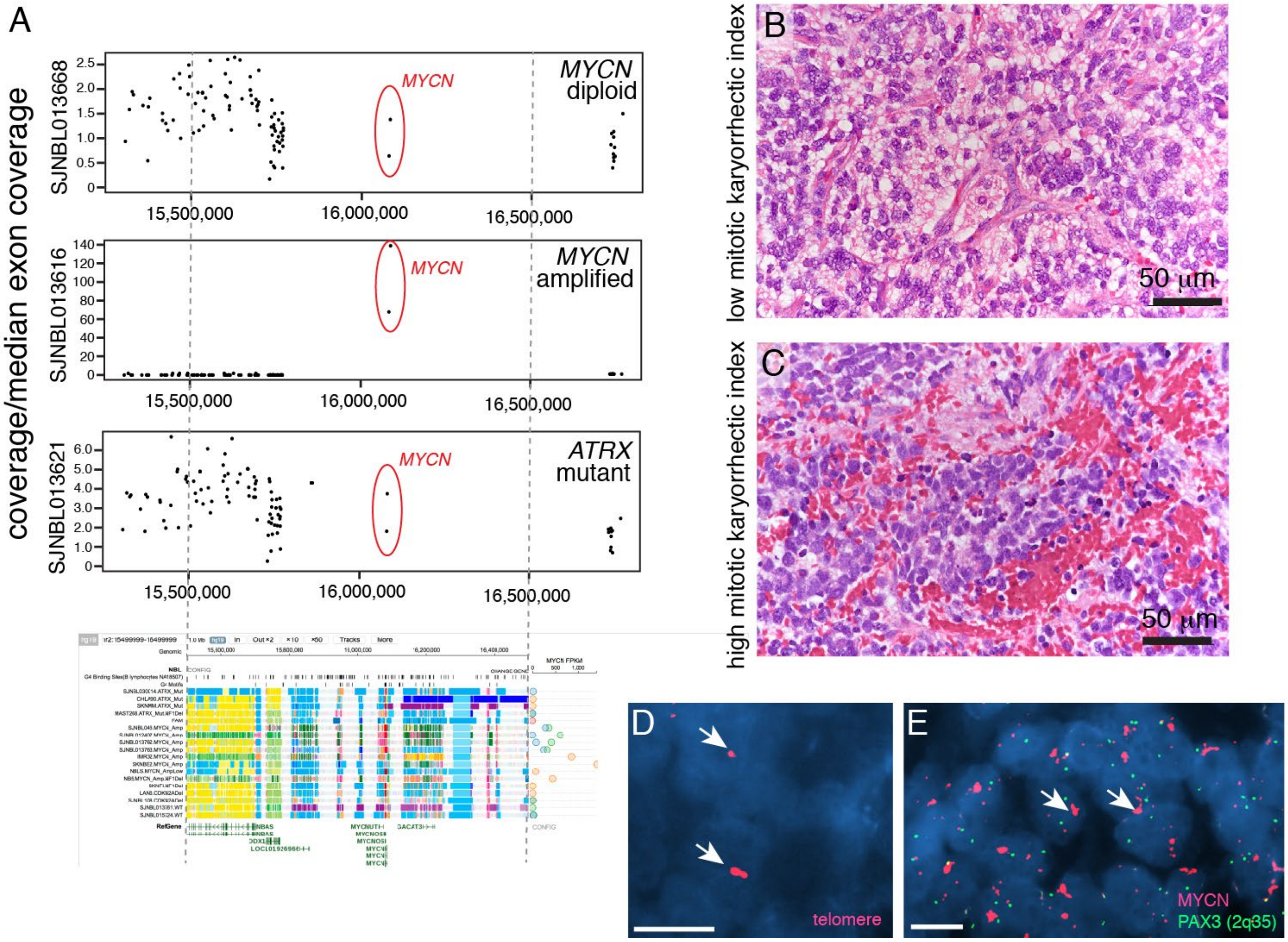

Supplementary Figure 1. Analysis of *MYCN* amplified *ATRX* mutant sample. A) Scatterplot of the coverage relative to the median exon coverage for the 3 indicated samples. SJNBL013668 has the normal diploid copy number of the MYCN locus, SJNBL013616 has high level amplification and SJNBL013621 is the sample reported to have MYCN amplification and *ATRX* mutation. The genomic region is aligned with the ChromHMM viewer that is part of this study showing the location of the exons being plotted. **B,C)** Micrograph of Hematoxylin and Eosin staining of the SJNBL013621 sample showing regions of low and high mitotic karyorrhectic index. **D)** Micrograph of fluorescent in situ hybridization (FISH) for telomeres (red) with nuclear stain (blue) showing regions of ultrabright signal indicative of alternative lengthening of telomeres (ALT). **E)** Micrograph of FISH for *MYCN* (red) and a control probe *(PAX3,* green). Arrows indicate strong FISH signals for telomeres and *MYCN*. Scale bars B&C 50 μm, D&E 5 μm.

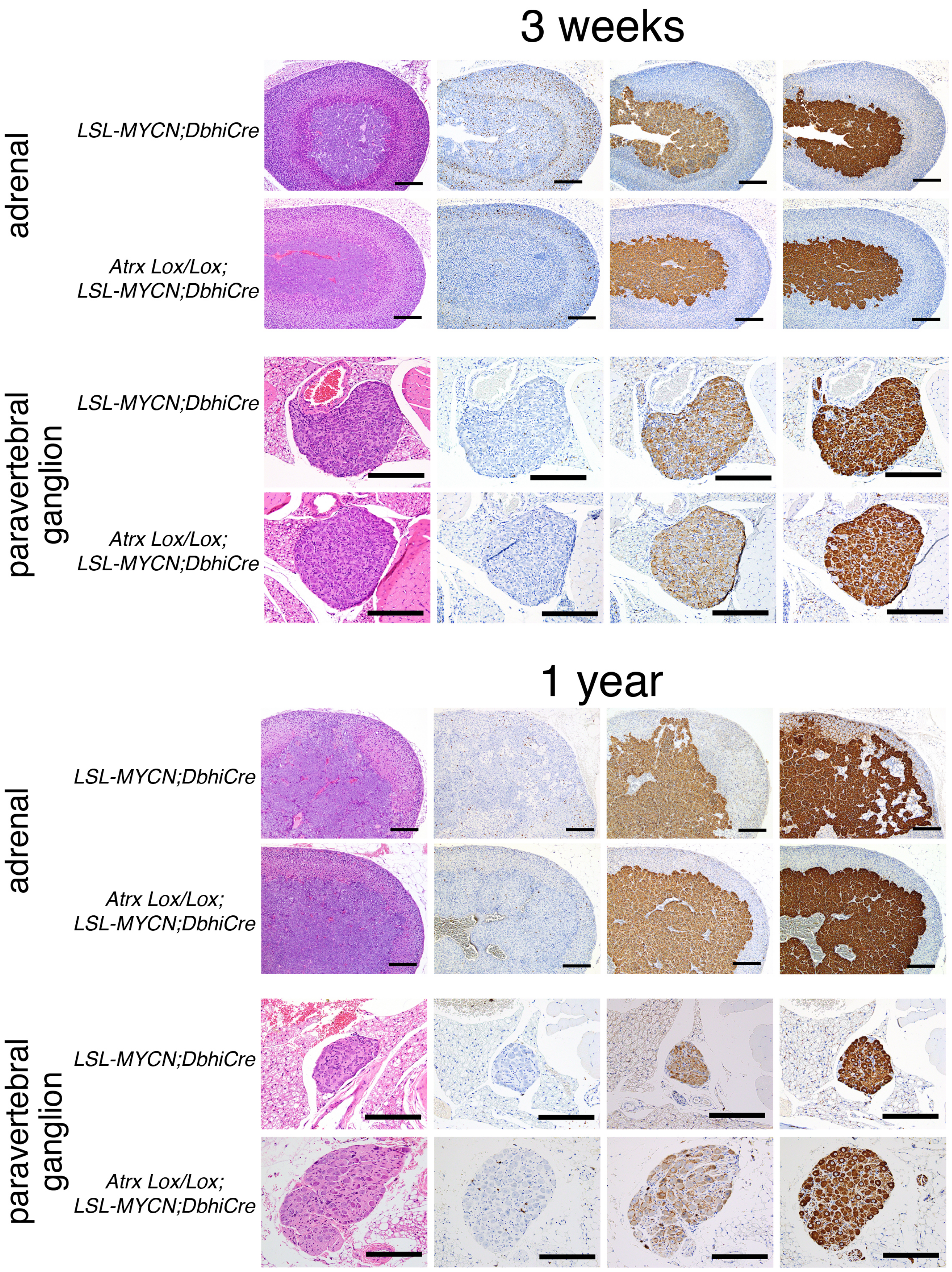

3 weeks
adrenal
LSL-MYCN;DbhiCre
Atrx Lox/Lox;
LSL-MYCN;DbhiCre
paravertebral
ganglion
LSL-MYCN;DbhiCre
Atrx Lox/Lox;
LSL-MYCN;DbhiCre
1 year
adrenal
LSL-MYCN;DbhiCre
Atrx Lox/Lox;
LSL-MYCN;DbhiCre
paravertebral
ganglion
LSL-MYCN;DbhiCre
Atrx Lox/Lox;
LSL-MYCN;DbhiCre

Supplementary Figure 2. Histopathologic review of genetically engineered mouse models. Micrographs of representative sections of adrenal and paravertebral ganglion from *Atrx*$^{Lox/Lox}$*;LSL-MYCN;DbhiCre* and control littermates (*LSL-MYCN;DbhiCre*). Hematoxylin and Eosin stained sections as well as Ki67, synaptophysin and tyrosine hydroxylase immunohistochemical staining are shown for each genotype at 3 weeks and 1 year of age. 3 animals for each genotype at each stage were reviewed by the veterinary pathologist in a blinded manner and no differences were identified. Scale bar: 100μm.

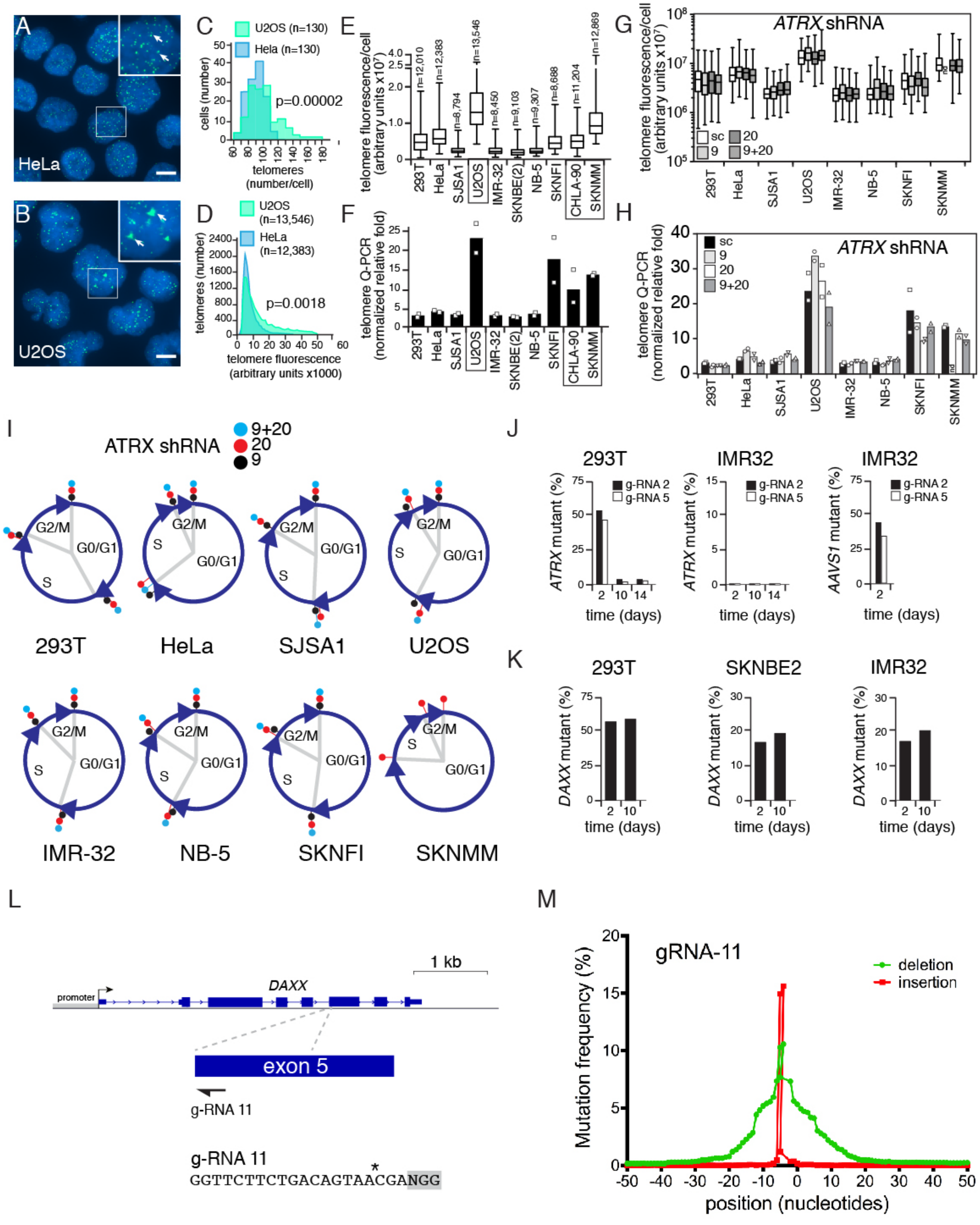
A
HeLa
B
U2OS
C
U2OS (n=130)
Hela (n=130)
cells (number)
p=0.00002
telomeres (number/cell)
D
U2OS (n=13,546)
HeLa (n=12,383)
telomeres (number)
p=0.0018
telomere fluorescence (arbitrary units x1000)
E
telomere fluorescence/cell (arbitrary units x10^7)
n=12,010
n=12,383
n=8,794
n=13,546
n=8,450
n=9,103
n=9,307
n=8,688
n=11,204
n=12,869
293T
HeLa
SJSA1
U2OS
IMR-32
SKNBE(2)
NB-5
SKNFI
CHLA-90
SKNMM
F
telomere Q-PCR (normalized relative fold)
G
ATRX shRNA
telomere fluorescence/cell (arbitrary units x10^7)
sc
9
20
9+20
H
ATRX shRNA
telomere Q-PCR (normalized relative fold)
I
ATRX shRNA
9+20
20
9
G2/M
G0/G1
S
293T
HeLa
SJSA1
U2OS
IMR-32
NB-5
SKNFI
SKNMM
J
293T
IMR32
IMR32
g-RNA 2
g-RNA 5
ATRX mutant (%)
AAVS1 mutant (%)
time (days)
K
293T
SKNBE2
IMR32
DAXX mutant (%)
time (days)
L
1 kb
promoter
DAXX
exon 5
g-RNA 11
g-RNA 11
GGTTCTTCTGACAGTAACGANGG
M
gRNA-11
deletion
insertion
Mutation frequency (%)
position (nucleotides)

Supplementary Figure 3. Inactivation of ATRX and DAXX. A,B) Micrograph of telomere FISH for HeLa and U2OS cells showing individual telomeres (arrows). The experiment was repeated with the same results. **C)** Step plot of the number of telomeres per cell for HeLa cells (blue) and U2OS cells (green). P value was calculated using Chi-square test **D)** Distribution of fluorescence intensity of individual telomeres in HeLa and U2OS cells. The ultrabright telomere signal in the U2OS (arrows in panel B) and higher intensity per telomere (green in D) are hallmarks of alternative lengthening of telomeres (ALT). P value was calculated using Chi-square test. **E)** Box and Whiskers plot of telomere fluorescence per cell for the indicated number of telomeres for each cell line. Each Box shows the 10th to 90th percentiles range of data, the line represents the median and the Whiskers show the minimum and maximum data range **F)** Bar plot with all data points presented of the amount of telomere DNA as measured by QPCR for each of the cell lines. The analysis was done twice while the graph shows the data from one experiment in a technical duplicate **G)** Box and Whiskers plot of telomere fluorescence per cell for each cell line transfected with the indicated *ATRX-shRNA* (130 cells were analyzed per condition). Each Box shows the 10th to 90th percentiles range of data, the line represents the median and the Whiskers show the minimum and maximum data range **H)** Bar plot with all data points presented of the amount of telomere DNA as measured by QPCR for each cell line transfected with the indicated *ATRX-shRNA* in technical duplicates. **I)** Piecharts of the cell cycle distribution for each of the shRNAs for each cell line. **J)** Proportion of MiSeq reads from the PCR product spanning the target sequence for g-RNA 2 and g-RNA 5 in 293T, SKNBE2 and IMR32 cells after 2, 10 and 14 days in culture. A control for IMR32 (AAVS1) is shown to demonstrate that efficient CRISPR/Cas9 targeting can be achieved in those cells. **K)** Proportion of MiSeq reads (with at least 200,000 reads) from the PCR product spanning the target sequence for g-RNA11 targeting exon 5 of DAXX. **L)** Map of the location of g-RNA 11 targeting exon 5 of DAXX. **M)** Plot of the frequency of mutation for g-RNA 2 with deletions in green and insertions in red from MiSeq analysis of the PCR product spanning the target sequence of DAXX. Scale bar: 5 μm.

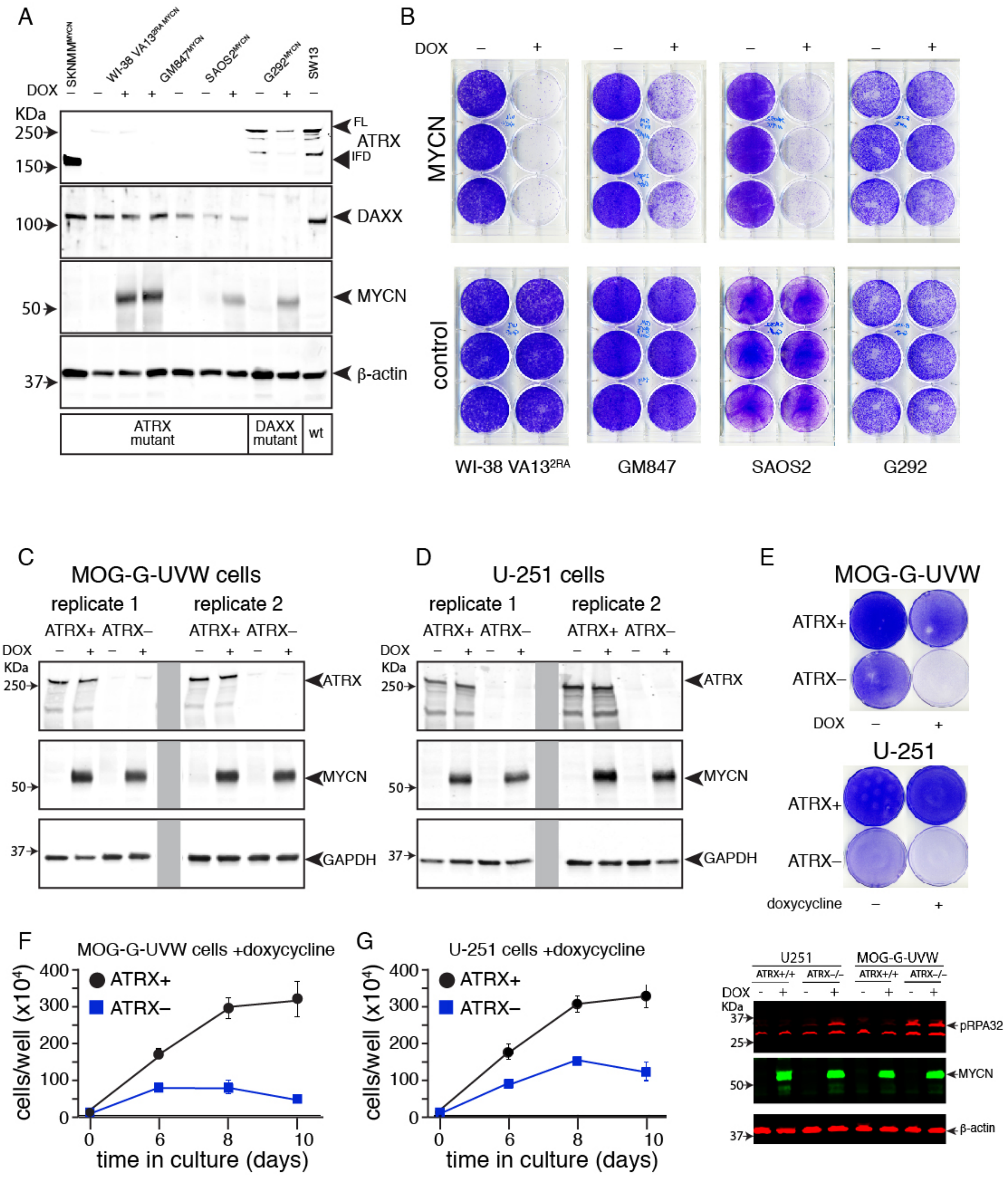
A
SKNMM^MYCN
WI-38 VA13^2RA MYCN
GM847^MYCN
SAOS2^MYCN
G292^MYCN
SW13
DOX
KDa
250
150
100
50
37
FL
ATRX
IFD
DAXX
MYCN
β-actin
ATRX mutant
DAXX mutant
wt
B
DOX
MYCN
control
WI-38 VA13^2RA
GM847
SAOS2
G292
C
MOG-G-UVW cells
replicate 1
replicate 2
ATRX+ ATRX–
ATRX+ ATRX–
DOX
KDa
250
50
37
ATRX
MYCN
GAPDH
D
U-251 cells
replicate 1
replicate 2
ATRX+ ATRX–
ATRX+ ATRX–
DOX
KDa
250
50
37
ATRX
MYCN
GAPDH
E
MOG-G-UVW
ATRX+
ATRX–
DOX
U-251
ATRX+
ATRX–
doxycycline
F
MOG-G-UVW cells +doxycycline
ATRX+
ATRX–
cells/well (x10^4)
time in culture (days)
G
U-251 cells +doxycycline
ATRX+
ATRX–
cells/well (x10^4)
time in culture (days)
U251
MOG-G-UVW
ATRX+/+
ATRX–/–
ATRX+/+
ATRX–/–
DOX
KDa
37
25
50
37
pRPA32
MYCN
β-actin

Supplementary Figure 4. Analysis of additional ATRX and DAXX deficient cell lines. A) Immunoblot of 6 cell lines that have stable integration of the doxycycline inducible MYCN expression plasmid. Lysates were prepared 4 days after addition of doxycycline and blotted for ATRX, DAXX, MYCN and β-actin as a loading control. SKNMM cells have an in frame deletion of ATRX (IFD), WI-38 $VA13^{2RA}$, GM847 and SAOS2 cells lack ATRX while G292 cells lack DAXX. The SW13 cells were included as a wild type control for ATRX and DAXX. The experiment was repeated with similar results **B)** Photograph of a cresyl violet stained 6 well dish from colony assays with each of the indicated cell lines in the presence and absence of doxycycline. The control lines have the tet regulated plasmid integrated into the genome but lacking the MYCN transgene. The experiment was repeated with similar results **C,D)** Immunoblot of 2 glioma cell lines with stable integration of the doxycycline inducible MYCN expression plasmid. Lysates were prepared 4 days after addition of doxycycline and blotted for ATRX, MYCN and GAPDH. **E)** Photograph of cresyl violet stained 6 well dish from colony assay with each line. The experiment was repeated with similar results. **F,G)** Line graph of growth curve for the indicated cell line with the MYCN inducible vector in the presence and absence of doxycycline. Each point is the mean and standard deviation of triplicate samples. **H)** Immunoblot for pRPA32 (red), MYCN (green) and an internal loading control (actin in red).

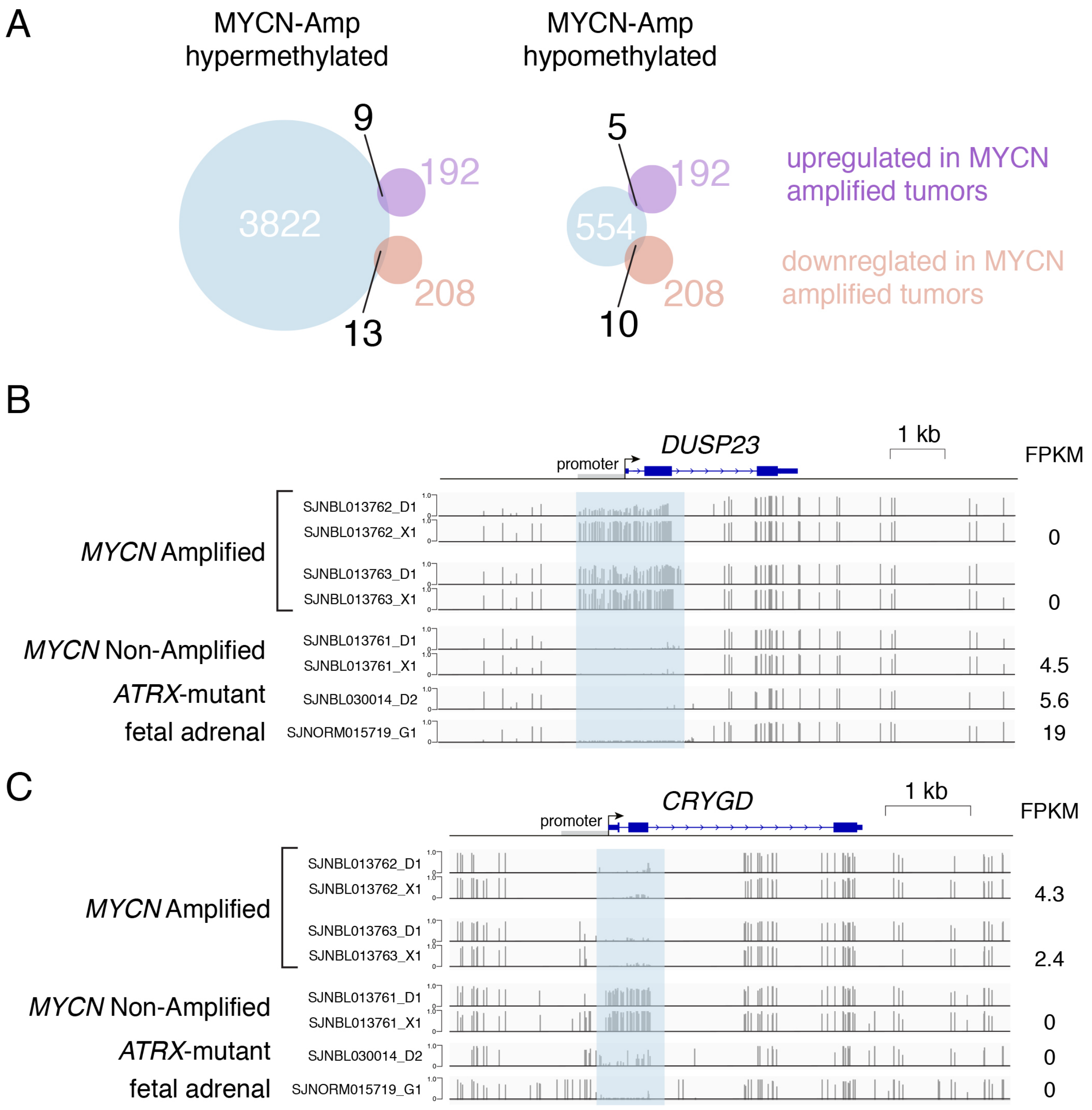
A
MYCN-Amp hypermethylated
MYCN-Amp hypomethylated
3822
9
192
208
13
554
5
192
208
10
upregulated in MYCN amplified tumors
downregleted in MYCN amplified tumors
B
DUSP23
promoter
1 kb
FPKM
MYCN Amplified
SJNBL013762_D1
SJNBL013762_X1
0
SJNBL013763_D1
SJNBL013763_X1
0
MYCN Non-Amplified
SJNBL013761_D1
SJNBL013761_X1
4.5
ATRX-mutant
SJNBL030014_D2
5.6
fetal adrenal
SJNORM015719_G1
19
C
CRYGD
promoter
1 kb
FPKM
MYCN Amplified
SJNBL013762_D1
SJNBL013762_X1
4.3
SJNBL013763_D1
SJNBL013763_X1
2.4
MYCN Non-Amplified
SJNBL013761_D1
SJNBL013761_X1
0
ATRX-mutant
SJNBL030014_D2
0
fetal adrenal
SJNORM015719_G1
0

Supplementary Figure 5. DNA methylation analysis. A) Venn diagram of the hypermethylated and hypomethylated promoters (blue) in the *MYCN* amplified tumors relative to the non-amplified tumors. The upregulated genes are shown in purple and the downregulated genes are shown in brown. The number of overlapping genes are indicated on the diagram. **B,C)** Representative plots of DNA methylation from whole genome bisulfite sequencing for a representative hypermethylated promoter (*DUSP23*) in *MYCN* amplified tumors that correlated with reduced gene expression (FPKM is shown to the right) and a hypomethylated promoter (*CRYGD)* that correlated with elevated gene expression. Abbreviations: FPKM, fragments per kilobase of transcript per million reads.

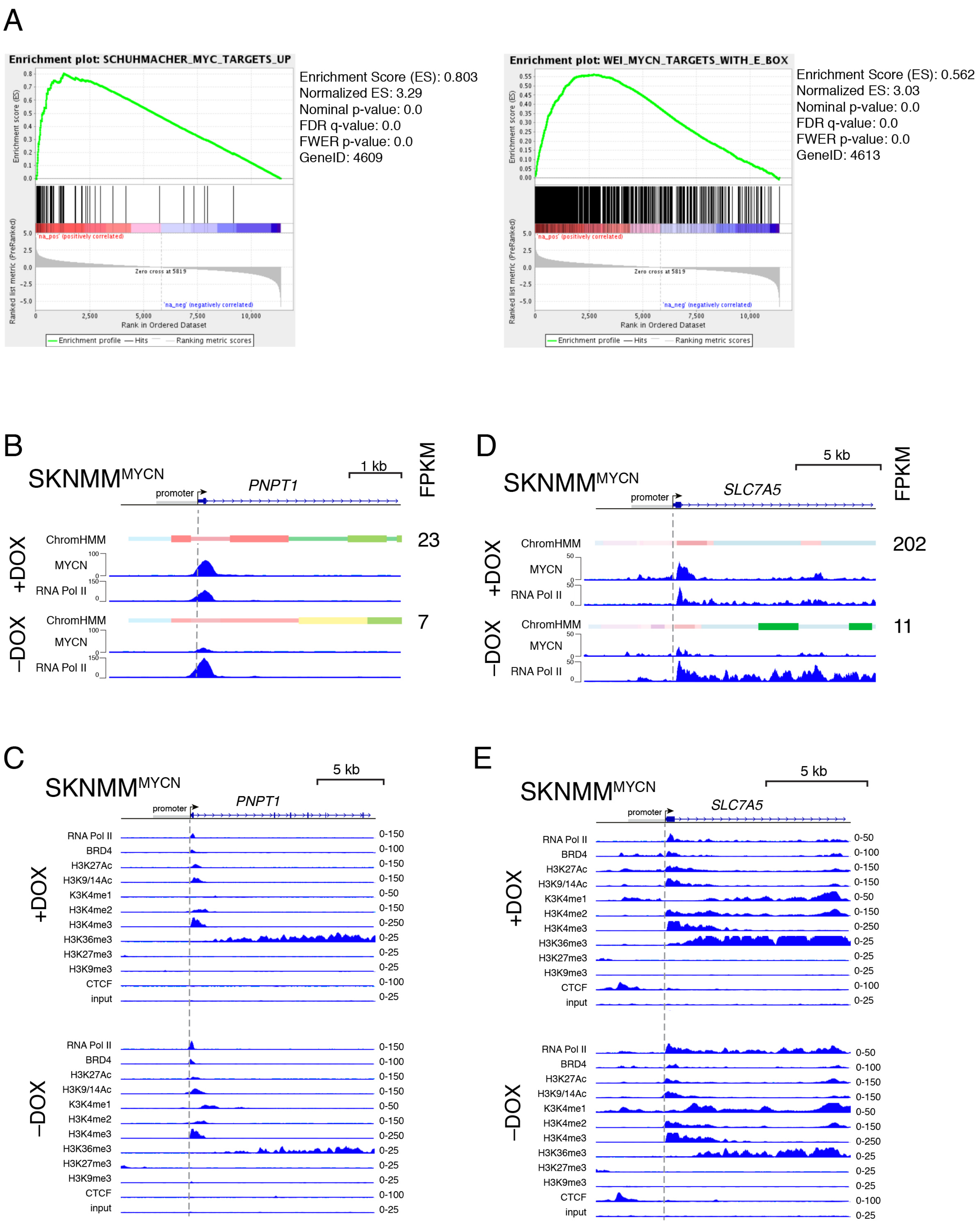
A
Enrichment plot: SCHUHMACHER_MYC_TARGETS_UP
Enrichment Score (ES): 0.803
Normalized ES: 3.29
Nominal p-value: 0.0
FDR q-value: 0.0
FWER p-value: 0.0
GeneID: 4609
Enrichment plot: WEI_MYCN_TARGETS_WITH_E_BOX
Enrichment Score (ES): 0.562
Normalized ES: 3.03
Nominal p-value: 0.0
FDR q-value: 0.0
FWER p-value: 0.0
GeneID: 4613
Enrichment score (ES)
Ranked list metric (PreRanked)
Zero cross at 5819
'na_pos' (positively correlated)
'na_neg' (negatively correlated)
Rank in Ordered Dataset
Enrichment profile
Hits
Ranking metric scores
B
SKNMM^MYCN
PNPT1
promoter
1 kb
FPKM
+DOX
ChromHMM
MYCN
RNA Pol II
23
–DOX
7
D
SLC7A5
5 kb
202
11
C
E
RNA Pol II
BRD4
H3K27Ac
H3K9/14Ac
K3K4me1
H3K4me2
H3K4me3
H3K36me3
H3K27me3
H3K9me3
CTCF
input
0-150
0-100
0-50
0-250
0-25

Supplementary Figure 6. Induction of MYCN targets in SKNMM^{MYCN} cells. A) The top pathways in gene set enrichment analysis for SKNMM^{MYCN} and U2OS^{MYCN} cells in the presence of doxycycline relative to the absence of doxycycline. **B)** ChIP-seq for MYCN and RNA Pol II and chrom-HMM for the *PNPT1* gene promoter in SKNMM^{MYCN} cells in the presence and absence of doxycycline. The PNPT1 protein is important for importing nuclear encoded RNA into the mitochondria. **C)** All ChIP-seq tracks for the *PNPT1* gene in the presence and absence of doxycycline. **D)** ChIP-seq for MYCN and RNA Pol II and chrom-HMM for the *SLC7A5* gene promoter in SKNMM^{MYCN} cells in the presence and absence of doxycycline. The SLC7A5 protein is important for glutamine transport. **E)** All ChIP-seq tracks for the *SLC7A5* gene in the presence and absence of doxycycline.

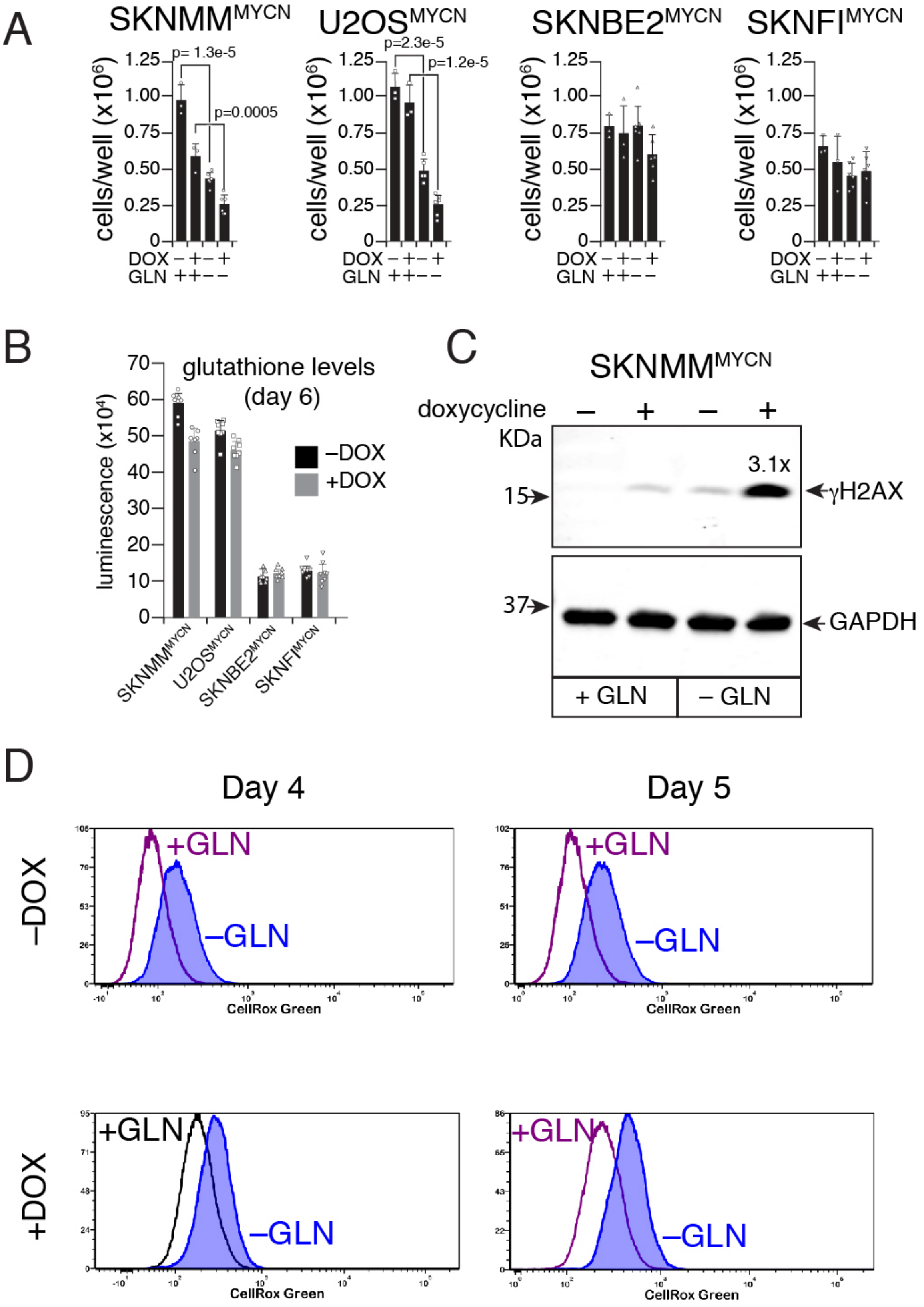
A
SKNMM^{MYCN}
U2OS^{MYCN}
SKNBE2^{MYCN}
SKNFI^{MYCN}
cells/well (x10^6)
p= 1.3e-5
p=0.0005
p=2.3e-5
p=1.2e-5
DOX − + − +
GLN + + − −
B
glutathione levels (day 6)
luminescence (x10^4)
−DOX
+DOX
C
SKNMM^{MYCN}
doxycycline − + − +
KDa
3.1x
15
γH2AX
37
GAPDH
+ GLN
− GLN
D
Day 4
Day 5
−DOX
+DOX
+GLN
−GLN
CellRox Green

Supplementary Figure 7. Glutamine metabolism following MYCN induction. A) Bar plot with individual data points presented of the scoring of the number of cells per well for each cell line cultured in the presence or absence of glutamine with and without doxycycline. Each bar is the mean and standard deviation of 3-6 wells. SKNMM and U2OS cells were significantly sensitive to glutamine deprivation, Student T-test) **B)** Bar plot with individual data points presented of glutathione levels in the cells on day 6 in culture for each cell line in the presence and absence of doxycycline. Each bar is the mean and standard deviation of 8 wells **C)** Immunoblot of γ-H2AX and GAPDH in the presence and absence of doxycycline and glutamine (GLN). The fold induction is indicated over the γ-H2AX band in the presence of doxycycline relative to the absence of doxycycline in the absence of glutamine. **D)** Flow cytometry of cells stained with CellRox Green to measure reactive oxygen species (ROS) on day 4 and day 5 in the presence and absence of doxycycline and glutamine.

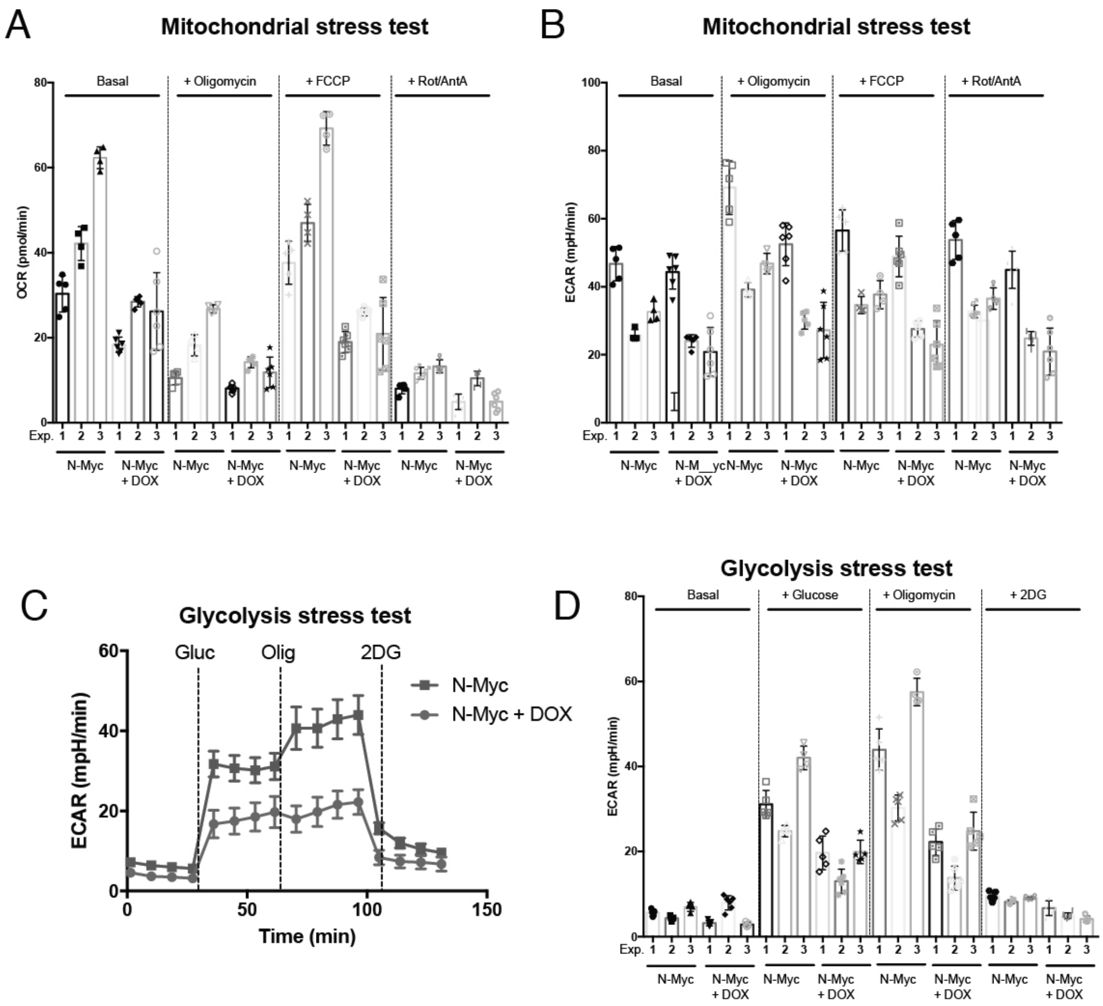
A
Mitochondrial stress test
Basal
+ Oligomycin
+ FCCP
+ Rot/AntA
OCR (pmol/min)
Exp.
N-Myc
N-Myc + DOX
B
Mitochondrial stress test
Basal
+ Oligomycin
+ FCCP
+ Rot/AntA
ECAR (mpH/min)
Exp.
N-Myc
N-Myc + DOX
C
Glycolysis stress test
Gluc
Olig
2DG
N-Myc
N-Myc + DOX
ECAR (mpH/min)
Time (min)
D
Glycolysis stress test
Basal
+ Glucose
+ Oligomycin
+ 2DG
ECAR (mpH/min)
Exp.
N-Myc
N-Myc + DOX

Supplementary Figure 8. Mitochondrial stress and glycolysis stress for SKNMM^{MYCN} cells. **A)** Bar plot with individual data points presented of 3 independent experiments showing the mean and standard deviation for each experiment and each sample at basal and treated conditions. The oxygen consumption rate is plotted in this panel. **B)** The extracellular acidification rate (ECAR) is plotted for the same samples as shown in (A). For the data in A and B, the experiment was performed using conditions for mitochondrial stress testing on the Seahorse instrument. **C)** Representative experiment out of three independent ones showing the ECAR for SKNMM^{MYCN} cells in the presence and absence of doxycycline grown under glycolysis stress conditions. Each point represents the average and the standard error of the mean for the technical replicates in the same experiment. **D)** Bar plot with individual data points presented of 3 independent experiments for the glycolysis stress test conditions for the indicated cell lines.

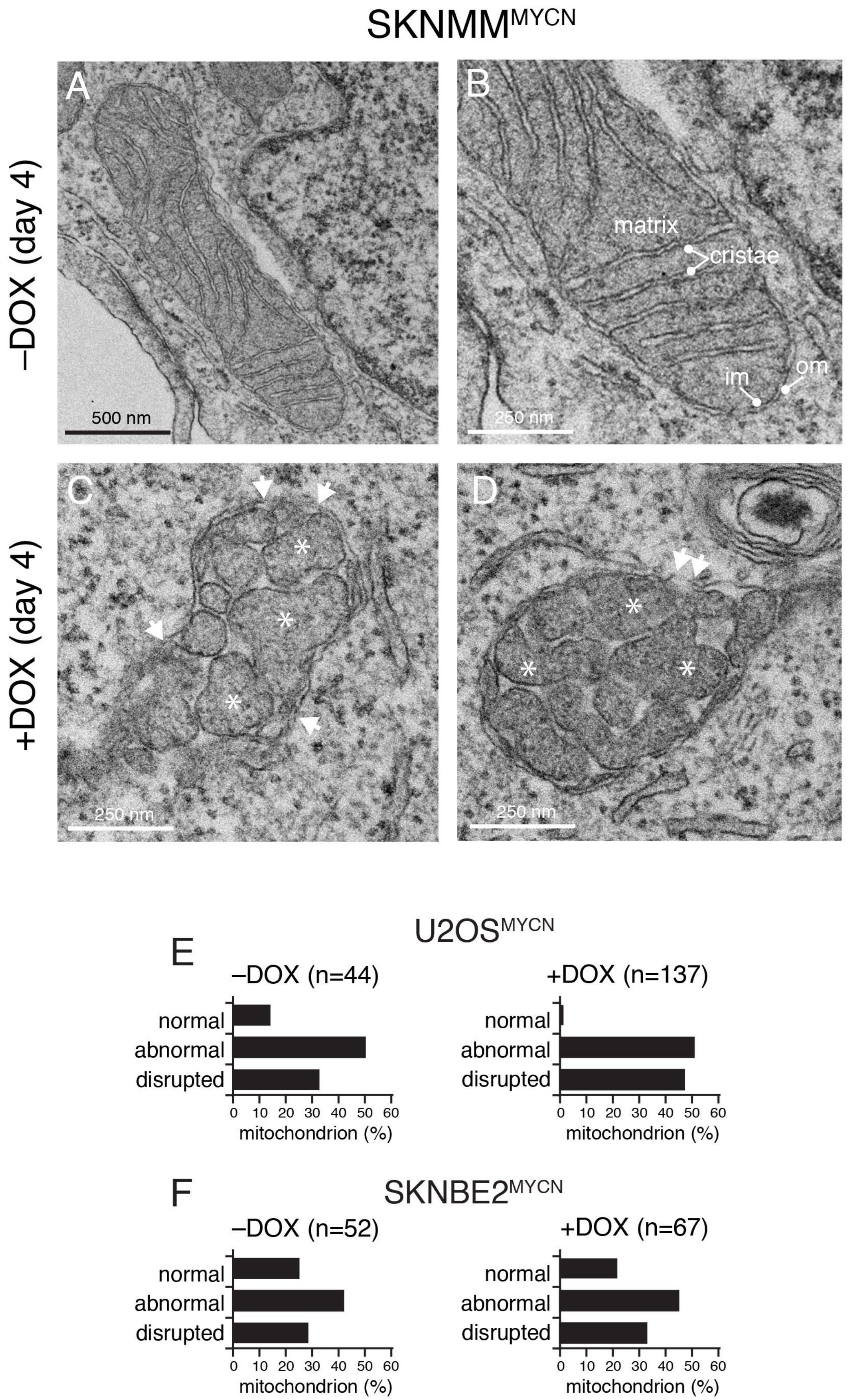
SKNMM^{MYCN}
A
B
–DOX (day 4)
matrix
cristae
im
om
500 nm
250 nm
C
D
+DOX (day 4)
250 nm
250 nm
U2OS^{MYCN}
E
–DOX (n=44)
+DOX (n=137)
normal
abnormal
disrupted
0 10 20 30 40 50 60
mitochondrion (%)
F
SKNBE2^{MYCN}
–DOX (n=52)
+DOX (n=67)
normal
abnormal
disrupted
0 10 20 30 40 50 60
mitochondrion (%)

Supplementary Figure 9. Electron microscopy of mitochondria in SKNMM^{MYCN} cells. A,B) Micrograph of SKNMM^{MYCN} cell mitochondria after 4 days in culture in the absence of doxycycline. The mitochondrial matrix and cristae are labeled as well as the inner and outer mitochondrial membranes (im and om). **C,D)** Micrograph of SKNMM^{MYCN} cell mitochondria after 4 days in culture in the presence of doxycycline. The cristae are disorganized and swollen and the mitochondrial membranes are disrupted (arrows). **E,F)** Bar plot of scoring of mitochondrial morphology for the U2OS^{MYCN} and SKNBE2^{MYCN} cells in the presence or absence of doxycycline after 4 days in culture.

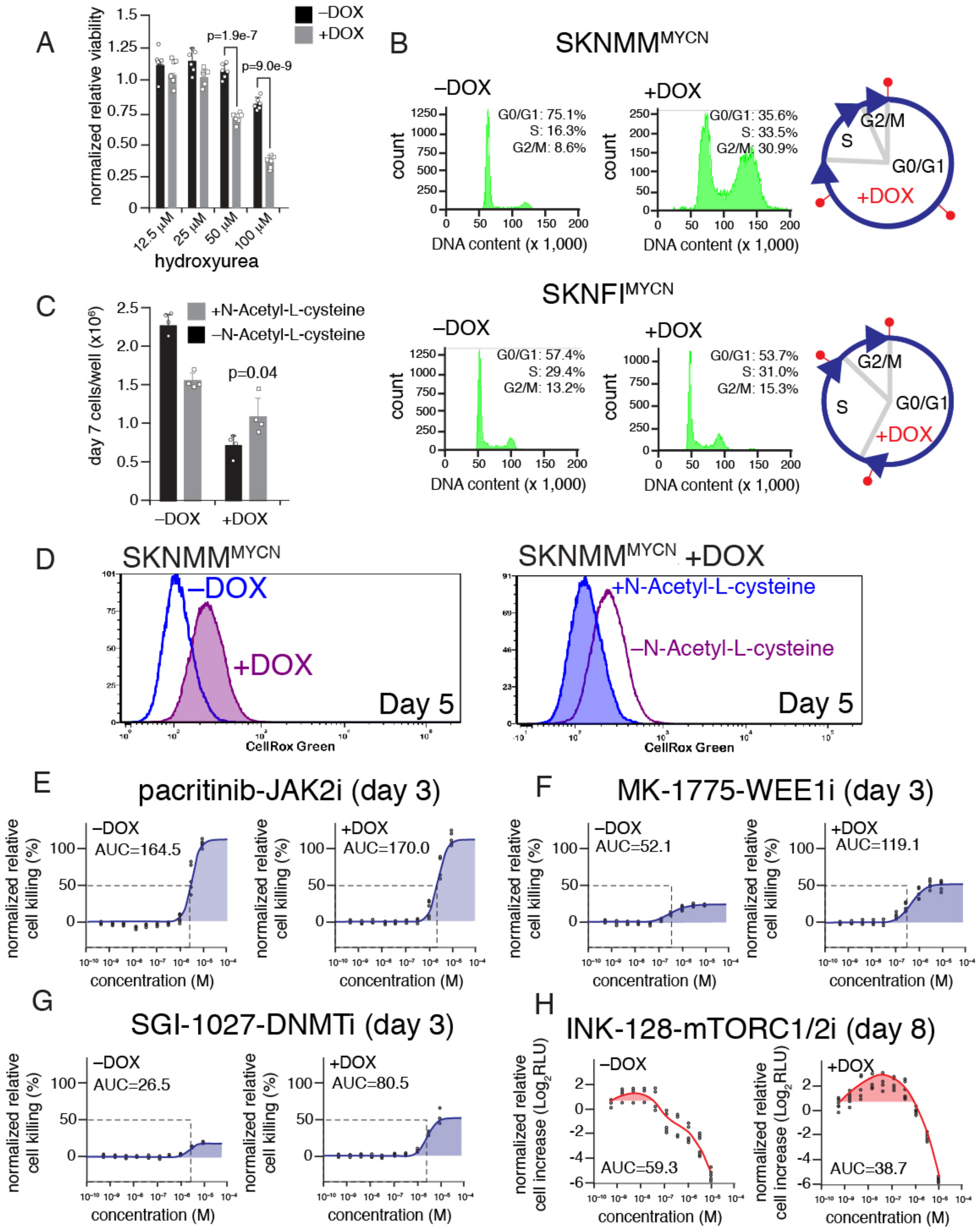

A
B
C
D
E
F
G
H
SKNMM^MYCN
SKNFI^MYCN
pacritinib-JAK2i (day 3)
MK-1775-WEE1i (day 3)
SGI-1027-DNMTi (day 3)
INK-128-mTORC1/2i (day 8)

Supplementary Figure 10. Oxidative stress contributes to synthetic lethality. A) Bar plot with individual data points presented of the relative viability of $SKNMM^{MYCN}$ cells in the presence and absence of doxycycline on day 4 in culture in the presence of different concentrations of hydroxyurea. Each bar is the mean and standard deviation of 8 wells. P-value was calculated using two-tailed Student T-test **B)** Plot of DNA content per cell and relative proportion of cells in G0/G1, S and G2/M phases of the cell cycle on day 4 in culture for $SKNMM^{MYCN}$ and $SKNFI^{MYCN}$ cells in the presence and absence of doxycycline. The piechart shows the proportion of each phase for the untreated (-DOX, gray lines) and in the presence of doxycycline (red). **C)** Bar plot with individual data points presented of $SKNMM^{MYCN}$ cells on day 7 after addition of doxycycline in the presence and absence of N-Acetyl-L-cysteine. Each bar is the mean and standard deviation of 4 different biologic replicates. P value was calculated using two-tailed Student T-test **D)** The same samples analyzed in (C) were also analyzed by flow cytometry using CellRox Green to measure reactive oxygen species (ROS). The panel on the left shows +/- doxycycline and the panel on the right shows +/- N-Acetyl-L-cysteine in the presence of doxycycline. **E-G)** Representative dose response curves for SKNMM cells on day 3 of culture in the presence and absence of doxycycline for a drug that had no effect on viability (F) and two drugs that potentiated killing my MYCN. The dashed line indicates the EC_{50} and the area under the curve is shown for each graph. **H)** Spline plot (see Supplemental Information) for one of two drugs that partially rescued the cell death induced by MYCN in SKNMM cells.

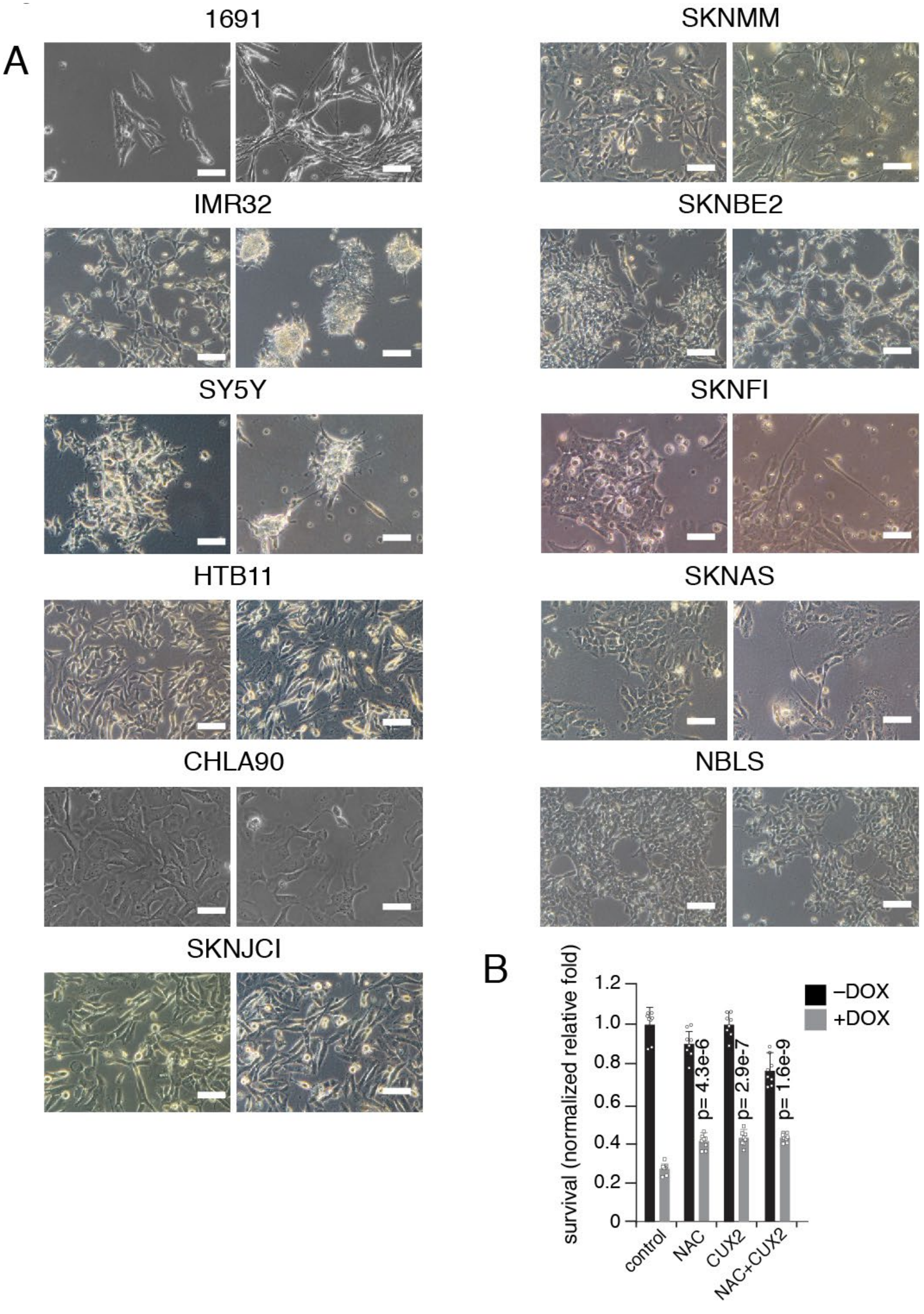
A
1691
SKNMM
IMR32
SKNBE2
SY5Y
SKNFI
HTB11
SKNAS
CHLA90
NBLS
SKNJCI
B
survival (normalized relative fold)
1.2
1.0
0.8
0.6
0.4
0.2
0
–DOX
+DOX
p= 4.3e-6
p= 2.9e-7
p= 1.6e-9
control
NAC
CUX2
NAC+CUX2

Supplementary Figure 11. Neuroblastoma differentiation and CUX2. A) Micrograph of 11 NB cell lines cultured in the presence and absence of retinoic acid for 6 days to induce differentiation. The same cultures were used for the RNA-seq analysis to determine if CUX2 was induced with differentiation. **B)** Bar plot with individual data points of the mean and standard deviation of cell survival measured with CellTiterGlo for $SKNMM^{MYCN}$ cells in the presence and absence of doxycycline (DOX). There was a significant increase in viability when NAC was added to the culture or CUX2 was ectopically expressed but no added benefit of the combination. P values were calculated using two-tailed Student T-test, n=8 biologic replicates. Scale bars 10 μm.

Figure 2A

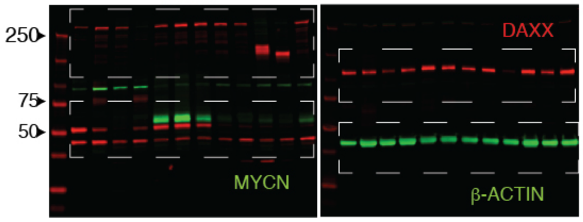

Figure 2H

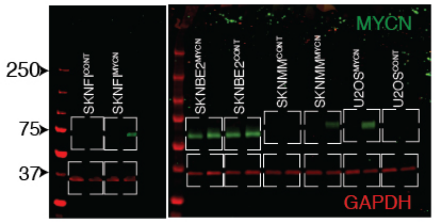

Figure 2B

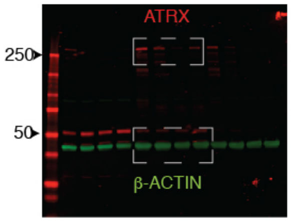

Figure 2M

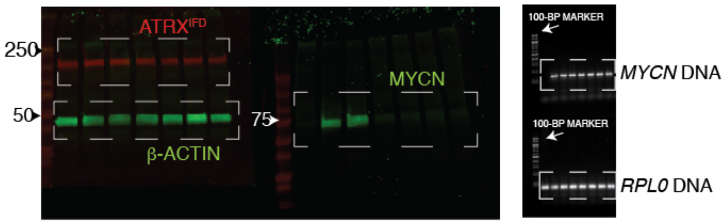

Figure 5B

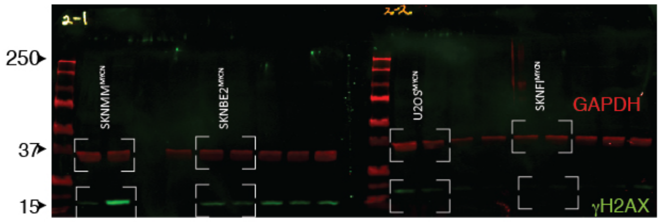

Figure 5I

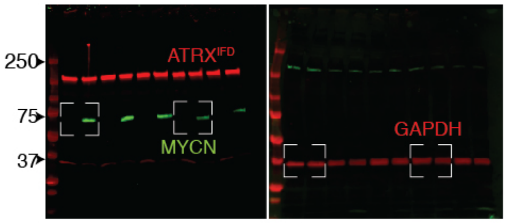

Figure S4A

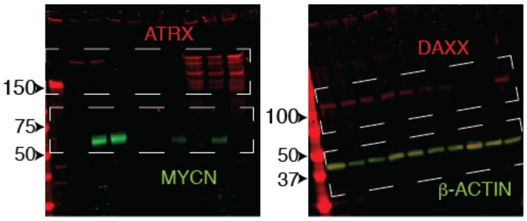

Figure S4H

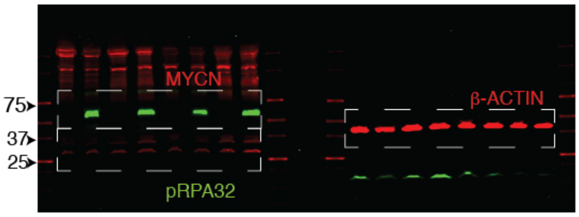

Figure S4C

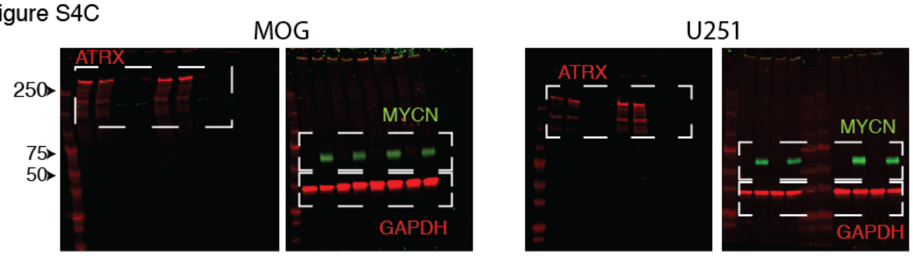

Figure S7C

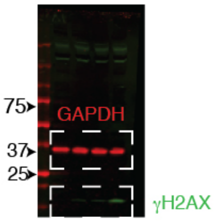

Supplementary Figure 12. Uncropped gels and blots presented in this paper. Original Western Blots and PCR gels used in this paper are shown here. The cropped regions are outlined by dashed white rectangles.

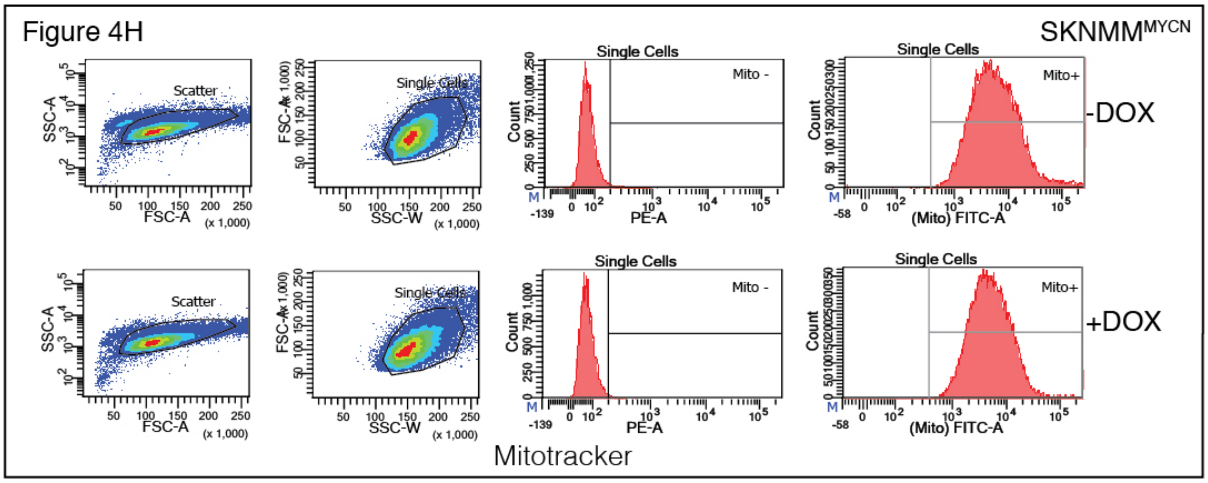
Figure 4H
SKNMM^MYCN
Scatter
Single Cells
Mito -
Mito+
-DOX
+DOX
SSC-A
FSC-A
SSC-W
Count
PE-A
(Mito) FITC-A
Mitotracker

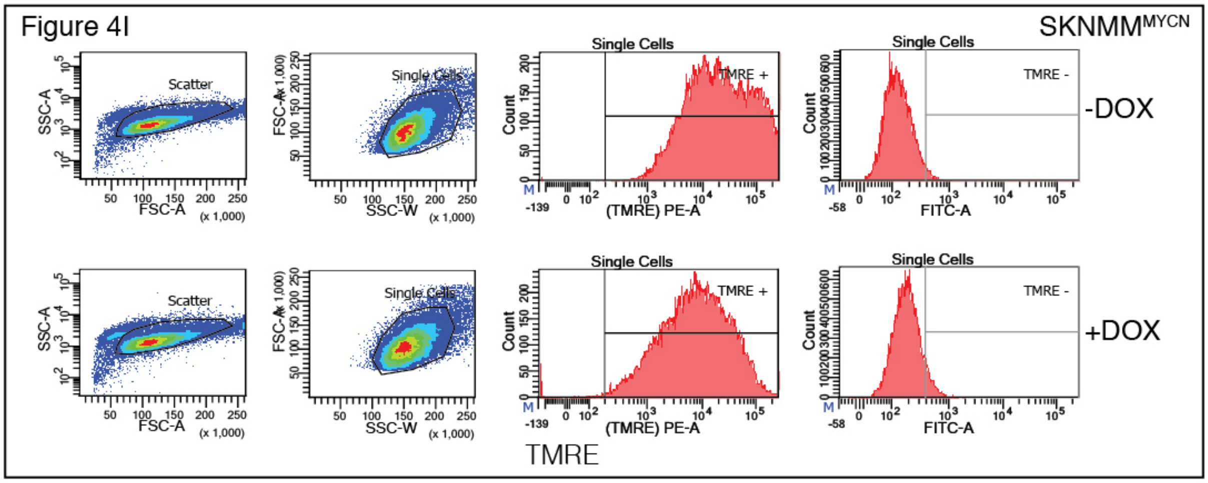
Figure 4I
SKNMM^MYCN
Scatter
Single Cells
TMRE +
TMRE -
-DOX
+DOX
SSC-A
FSC-A
SSC-W
Count
(TMRE) PE-A
FITC-A
TMRE

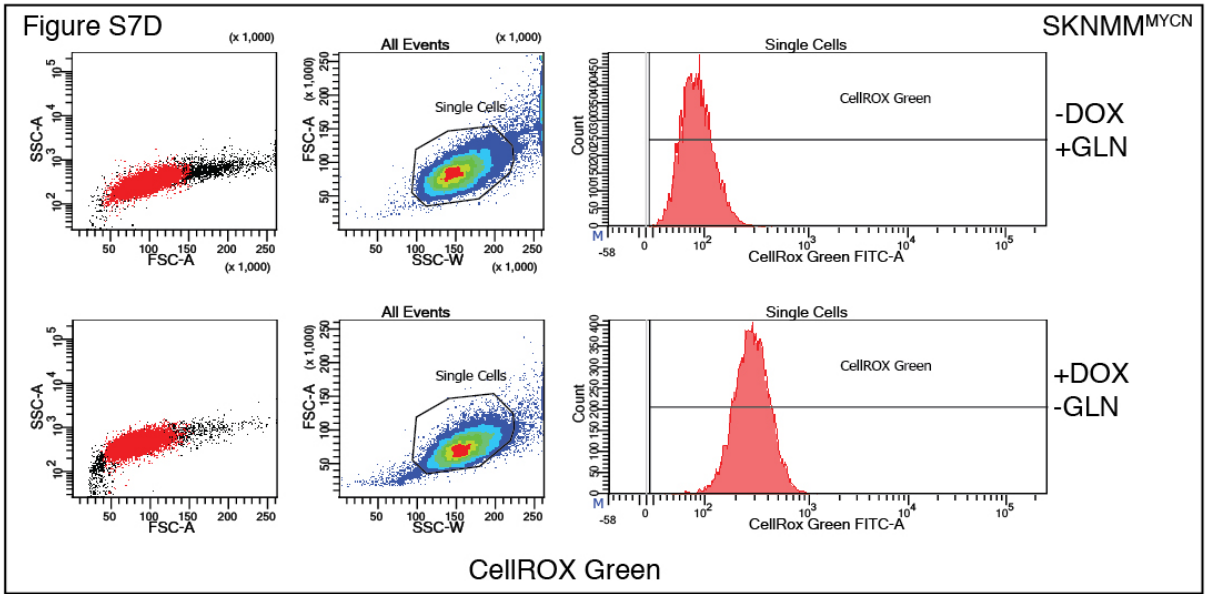
Figure S7D
SKNMM^MYCN
All Events
Single Cells
CellROX Green
-DOX
+GLN
+DOX
-GLN
SSC-A
FSC-A
SSC-W
Count
CellRox Green FITC-A
CellROX Green

Supplementary Figure 13: Flowcytometry analysis and gating. Left 2 panels show the gating strategies used for flowcytometric analysis. Right panels show representative histograms for different fluorescent channels used in this study.

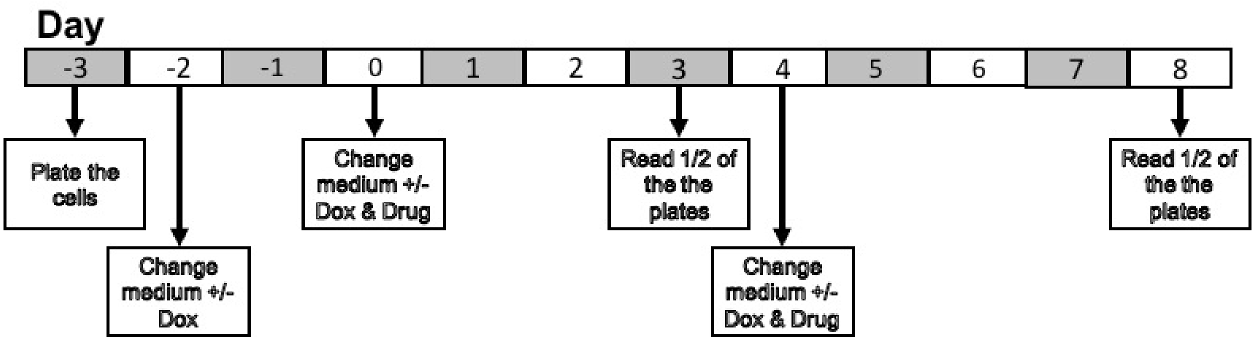
Day
-3
-2
-1
0
1
2
3
4
5
6
7
8
Plate the cells
Change medium +/- Dox
Change medium +/- Dox & Drug
Read 1/2 of the the plates
Change medium +/- Dox & Drug
Read 1/2 of the the plates

Supplementary Figure 14: Schematic representation of the dose response curve drug screening.

SUPPLEMENTARY NOTES

SUPPLEMENTARY NOTE 1

Testing mutual exclusivity between *ATRX*-mutations and *MYCN* amplification

To test the mutual exclusivity between *ATRX*-mutations and *MYCN* amplification in neuroblastoma, we merged the COG, TARGET, and PCGP data.

Below are results of the analysis looking at the relationship between *ATRX* and *MYCN* in the subgroup of patients with Stage 4, >5 years of age:

Variable	*N (%)*	*ATRX mutation (%)*	*p-value*
MYCN (investigator data)			
Not amplified	*153 (90.0)*	*41 (26.8)*	*0.0135*
Amplified	*17 (10.0)*	*0 (0)*	

If we look just at Stage 4, 5-12 years of age:

Variable	*N (%)*	*ATRX mutation (%)*	*p-value*
MYCN (investigator data)			
Not amplified	*113 (88.3)*	*25 (22.1)*	*0.0414*
Amplified	*15 (11.7)*	*0 (0)*	

We also looked at stratified analyses using the Cochrane-Mantel-Haenszel test. Stratifying/controlling by age (<18 months, 18 months-5 years, 5-12 years, >12 years), a significant difference was found in the proportion of *ATRX* mutations in the MYCN non-amplified vs. amplified groups, using *MYCN* combined COG, TARGET, PCGP data (p=0.0037).

ATRX mutations by *MYCN* – Stratified by Age

**Age group**	***ATRX* mutation**	***MYCN* (Investigator data)**	
		**Non-Amplified**	**Amplified**
**<18 months**	**No**	203	28

	**Yes**<br>**Percent Yes**	0<br>0%	0<br>0%
**18 months-5 years**	**No**<br>**Yes**<br>**Percent Yes**	191<br>13<br>6.37%	82<br>0<br>0%
**5-12 years**	**No**<br>**Yes**<br>**Percent Yes**	183<br>27<br>12.86%	16<br>0<br>0%
**>12 years**	**No**<br>**Yes**<br>**Percent Yes**	50<br>18<br>26.47%	2<br>0<br>0%

Stratifying/controlling by stage (non-Stage 4, Stage 4), a significant difference was found in the proportion of *ATRX* mutations in the *MYCN* non-amplified vs. amplified groups, using *MYCN* investigator data (p<0.0001).

***ATRX* mutations by *MYCN* – Stratified by Stage**

**Stage group**	***ATRX* mutation**	***MYCN* (Investigator data)**	
		**Non-Amplified**	**Amplified**
**Non-Stage 4**	**No**<br>**Yes**<br>**Percent Yes**	304<br>5<br>1.62%	3<br>0<br>0%
**Stage 4**	**No**<br>**Yes**<br>**Percent Yes**	323<br>53<br>14.1%	125<br>0<br>0%

Stratifying/controlling by age*stage (non-Stage 4, 18 months-5 years; Stage 4, 18 months-5 years; non-Stage 4, >5 years; Stage 4, >5 years), a significant difference was found in the proportion of ATRX mutations in the *MYCN* non-amplified vs. amplified groups, using *MYCN* COG, TARGET, PCGP data (p=0.0002).

***ATRX* mutations by *MYCN* – Stratified by Stage*Age**

**Stage*Age group**	***ATRX* mutation**	***MYCN* (Investigator data)**	
		**Non-Amplified**	**Amplified**
**Non-Stage 4, 18 months-5 years**	**No**	73	2
	**Yes**	1	0
	**Percent Yes**	1.35%	0%
**Stage 4, 18 months-5 years**	**No**	118	80
	**Yes**	12	0
	**Percent Yes**	9.23%	0%
**Non-Stage 4, >5 years**	**No**	121	1
	**Yes**	4	0
	**Percent Yes**	3.2%	0%
**Stage 4, >5 years**	**No**	112	17
	**Yes**	41	0
	**Percent Yes**	26.8%	0%

Supplementary Note 2

Histopathologic Review of Genetically Engineered Mouse Models

The exact mechanism by which MYCN induces neuroblastoma is not completely known. Hansford *et al*. showed that *TH-MYCN* mice exhibit areas of hyperplasia in the sympathetic ganglia[1]. Unlike wildtype mice, these hyperplastic areas persist beyond the first week after birth *TH-MYCN* mice and could be detected up to the age of 6 weeks. We could not detect similar hyperplastic regions at the age of 3 weeks in either *LSL-MYCN:Dbh-iCre* nor *Atrx^{flox}-LSL-MYCN:Dbh-iCre* mice. These results may underline differences between different neuroblastoma mouse models. Interestingly, *TH-MYCN* mice develop neuroblastoma mainly in paravertebral ganglia while, similar to human patients, adrenal glands are the predominant site of neuroblastoma in *LSL-MYCN:Dbh-iCre* mice. In this study, we examined both adrenal medullae and paravertebral ganglia in *LSL-MYCN:Dbh-iCre* and *Atrx^{flox}-LSL-MYCN:Dbh-iCre* mice. The data were reviewed by a veterinary pathologist and no differences were identified..

Supplementary references

1 Hansford, L. M. *et al.* Mechanisms of embryonal tumor initiation: distinct roles for MycN expression and MYCN amplification. *Proceedings of the National Academy of Sciences of the United States of America* **101**, 12664-12669, doi:10.1073/pnas.0401083101 (2004).
