# Supplementary material for: MYCN amplification and ATRX mutations are incompatible in neuroblastoma
Source: Nat Commun. 2020 Feb 14;11:913. doi: 10.1038/s41467-020-14682-6 (PMC7021759; doi:10.1038/s41467-020-14682-6)
Supplement: Supplementary file 3 — Description of Additional Supplementary Files [file 41467_2020_14682_MOESM3_ESM.pdf]

Description of Additional Supplementary Files

File Name: Supplementary Data 1
Description: Event-free survival, overall survival and hazard ratio for patients with *ATRX*-mutant neuroblastoma.

File Name: Supplementary Data 2
Description: Characteristics of cell lines and orthotopic patient-derived xenografts (O-PDX) used in this study.

File Name: Supplementary Data 3
Description: Quantitation of timing between cell death from the previous cytokinesis for $SKNMM^{MYCN}$ + doxycycline.

File Name: Supplementary Data 4
Description: Xenogen signal intensity of in-vivo orthotopic xenografts from $SKNMM^{MYCN}$ and $SKNMM^{CONT}$ cells.

File Name: Supplementary Data 5
Description: Analysis of differentially methylated regions (DMRs) in *MYCN*-amplified relative to *MYCN* non-amplified neuroblastoma.

File Name: Supplementary Data 6
Description: ChromHMM and core regulatory circuit (CRC) analyses of neuroblastoma.

File Name: Supplementary Data 7
Description: Gene expression and pathway analyses of *ATRX*-mutant cell lines $SKNMM^{MYCN}$ and $U2OS^{MYCN}$ 4 days after *MYCN* induction.

File Name: Supplementary Data 8
Description: Quantification of ^{13}C-glucose-derived and ^{13}C-glutamine-derived metabolites in *ATRX*-mutant neuroblastoma cells and medium 4 days after *MYCN* induction.

File Name: Supplementary Data 9
Description: Dose response curve of drug screenings in *ATRX*-mutant neuroblastoma cells with *MYCN* induction.

File Name: Supplementary Data 10
Description: Gene expression analysis of neuroblastoma cell lines treated with retinoic acid (RA).

File Name: Supplementary Data 11
Description: Pathway analysis of H3.3 peaks depleted in *ATRX*-mutant neuroblastoma cells that correlate with ChromHMM.
