# Supplementary material for: MYCN amplification and ATRX mutations are incompatible in neuroblastoma
Source: Nat Commun. 2020 Feb 14;11:913. doi: 10.1038/s41467-020-14682-6 (PMC7021759; doi:10.1038/s41467-020-14682-6)
Supplement: Supplementary file 15 — Reporting Summary [file 41467_2020_14682_MOESM15_ESM.pdf]

nature research

Corresponding author(s): Michael A. Dyer

Last updated by author(s): 12/1/2019

Reporting Summary

Nature Research wishes to improve the reproducibility of the work that we publish. This form provides structure for consistency and transparency in reporting. For further information on Nature Research policies, seeAuthors & Referees and theEditorial Policy Checklist .

Statistics

For all statistical analyses, confirm that the following items are present in the figure legend, table legend, main text, or Methods section.

n/a	Confirmed	
[ ]	[x]	The exact sample size (*n*) for each experimental group/condition, given as a discrete number and unit of measurement
[ ]	[x]	A statement on whether measurements were taken from distinct samples or whether the same sample was measured repeatedly
[ ]	[x]	The statistical test(s) used AND whether they are one- or two-sided<br>*Only common tests should be described solely by name; describe more complex techniques in the Methods section.*
[ ]	[x]	A description of all covariates tested
[x]	[ ]	A description of any assumptions or corrections, such as tests of normality and adjustment for multiple comparisons
[ ]	[x]	A full description of the statistical parameters including central tendency (e.g. means) or other basic estimates (e.g. regression coefficient) AND variation (e.g. standard deviation) or associated estimates of uncertainty (e.g. confidence intervals)
[ ]	[x]	For null hypothesis testing, the test statistic (e.g. *F*, *t*, *r*) with confidence intervals, effect sizes, degrees of freedom and *P* value noted<br>*Give P values as exact values whenever suitable.*
[x]	[ ]	For Bayesian analysis, information on the choice of priors and Markov chain Monte Carlo settings
[x]	[ ]	For hierarchical and complex designs, identification of the appropriate level for tests and full reporting of outcomes
[x]	[ ]	Estimates of effect sizes (e.g. Cohen's *d*, Pearson's *r*), indicating how they were calculated

Our web collection on statistics for biologists contains articles on many of the points above.

Software and code

Policy information about availability of computer code

Data collection	MSigDB 6.1: Gene Set Enrichment analysis
Data analysis	ImageJ32: Comet Assay scoring<br>Graphpad Prism 6: Statistics<br>Live Imaging 4.5: Luciferase in mice (xenogen signal)<br>Softwares used to analyze ChIP Seq are described in ChIP-Seq report<br>ScanView software version 7.2.7: analysis FISH scan<br>CRISPResso 1.0.0: detecting the abundance of CRISPR-inducing NHEJ<br>CONSERTING 1.0: detecting copy number variation in whole genome sequencing<br>CREST 1.0: detecting structural variation in whole genome sequencing<br>MACS2 2.0.10.20131216: peak calling for CUT&RUN<br>Voom: statiscal analysis for CUT&RUN data<br>BSMAP2.74: alignment of bisulfite sequencing<br>R 3.3.2: statistical analysis WES, WGBS, Dose response curve<br>GENCODE (v26lift37): gene annotation RNAseq<br>ChromHMM 1.10: Hidden Markov Modeling of ChIP Seq data<br>ImageStudio Lite software 5.2.5: band analysis of Western blot<br>MasterHands 2.17.1.11: extract peaks for the metabolomics

For manuscripts utilizing custom algorithms or software that are central to the research but not yet described in published literature, software must be made available to editors/reviewers. We strongly encourage code deposition in a community repository (e.g. GitHub). See the Nature Research guidelines for submitting code & software for further information.

Data

Policy information about availability of data

All manuscripts must include a data availability statement. This statement should provide the following information, where applicable:

- Accession codes, unique identifiers, or web links for publicly available datasets
- A list of figures that have associated raw data
- A description of any restrictions on data availability

Whole-genome bisufite sequencing data and ChIP-seq data have been deposited in the EMBL-EBI database under the accession code EGAS00001003257 [https://www.ebi.ac.uk/ega/studies/EGAS00001003257]. Whole-exome sequencing data have been deposited in the EMBL-EBI database under the accession code EGAS00001002528 [https://www.ebi.ac.uk/ega/studies/EGAS00001002528]. All of our extensive epigenetic data and analysis are freely available in a cloud-based viewer [https://pecan.stjude.cloud/proteinpaint/study/mycn_nbl_2018]. All O-PDX tumors described here are freely available with no obligation to collaborate through the Childhood Solid Tumor Network [http://www.stjude.org/CSTN/]. We downloaded G4 Motifs from supplementary data of Du et al 200972 We downloaded 169,222 R-Loop domains in genic and proximal regions from [http://rloop.bii.a-star.edu.sg/]. All the other data supporting the findings of this study are available within the article and its supplementary information files and from the corresponding author upon reasonable request.

Field-specific reporting

Please select the one below that is the best fit for your research. If you are not sure, read the appropriate sections before making your selection.

[x] Life sciences [ ] Behavioural & social sciences [ ] Ecological, evolutionary & environmental sciences

For a reference copy of the document with all sections, see nature.com/documents/nr-reporting-summary-flat.pdf

Life sciences study design

All studies must disclose on these points even when the disclosure is negative.

Sample size

The first study that identified the presence of ATRX mutations in neuroblastoma was a comprehensive genetic analyses of stage 4 neuroblastoma tumors from infants (0-18 months of age), children (18 months-12 years of age) and adolescents and young adults (>12 years of age). In that study, we discovered that the ATRX gene is mutated in ~ 40% of adolescents and young adults, 17% of children and 0% of infants (Cheung N.K. et al JAMA, 2012). Importantly, the children with ATRX mutations tended to be older than 5 years of age and often had an indolent or chronic disease course similar to adolescents and young adults (AYA). These data led to the hypothesis that ATRX mutations are associated with an older age at diagnosis, a chronic/indolent disease course and overall poor outcome. However, limitations in the original data set included exclusive evaluation of stage 4 tumors, possible sample bias with tumors obtained from a single institution and a small sample size. Therefore the current study was performed to validate the initial study findings using multicenter samples collected under the leadership of the Children's Oncology Group (COG). Specifically, the goal was to narrow the confidence interval (CI) for the prevalence of ATRX mutations present in neuroblastoma and to define the prognostic significance of ATRX mutations.

The neuroblastoma sample sizes were determined in an attempt to narrow the 80% C.I. to approximately 10%. Further, all requested tumor sample sizes were selected to grossly reflect a representative sample of disease (neuroblastoma) stage by age group. For example in the previous study, the proportion of ATRX mutations present in stage 4 tumors of the 18mo – 5yrs age group was 17% with an 80% C.I. range of 5% - 33%. For this analysis, we estimated that we needed 100 stage 4 (metastatic) samples in this age group to achieve a C.I. of approximately 10%. In the previous study, we did not evaluate for ATRX mutations in non-metastatic or 4S disease. Therefore, the requested sample sizes for non-metastatic disease were based on the number of metastatic samples requested and were calculated to grossly reflect a representative sample of disease stage by age group. For example, approximately 60% of patients with neuroblastoma in the 18mo –5yrs age group are diagnosed with stage 4 (metastatic) disease and 40% are diagnosed with stages 1-3 (loco-regional) disease. Given that we estimated a need for 100 metastatic samples in this age group, in order to obtain a representative sample of disease stage by age group, we estimated that we needed 80 stages 1-3 (locoregional) samples.

The request was made to the Children's Oncology Group Neuroblastoma Committee and the cohort used in this analysis consists of 477 neuroblastoma patients (of which 476 had clinical data and 475 had outcome data available) representing a representative sample of risk levels, disease stages, and ages at diagnosis. All are enrolled on COG protocol ANBL12B8 ("Analysis of ATRX Mutations in Neuroblastoma") and were assayed for ATRX mutations. Roughly 80% of the patients were diagnosed between 2010 and 2012, with the remaining 20% diagnosed between 2001 and 2009.

Data exclusions

Samples lacked outcome data and 1 sample lacked clinical data. the criteria for data exclusion were per-established. It was believed that all samples had clinical and outcome data at the time that samples were randomly selected from the database. However, upon review, it was determined that a few samples lacked all corresponding data. Therefore, these samples were excluded.
In genetic mouse models, we censored out mice died for reasons other than tumors. The Criteria for data exclusion were per-established. Only mice that reached the survival age or died or sacrificed by a tumor were analyzed.

Replication

We have done every effort to ensure reproducibility of our data by, when possible, repeating the experiments using independent samples, including positive and negative controls and using multiple approaches to confirm our observations. We stated the sample number for each experiment and how many times each experiment was repeated in the figure legends.

Replication and validation by other assays were done whenever possible in this study:
-ATRX mutations and MYCN amplification was assessed by whole exome sequencing and custom capture and Illumina sequencing
-MYCN amplification status was assessed both by the neuroblastoma reference lab as part of ANBL00B1 enrollment and by St. Jude Children's

Research Hospital by 2 methods FISH and custom capture and Illumina sequencing
- ChIP Seq antibodies were previously validated and the validation procedure and data are available through St. Jude Childhood Solid Tumor Network website (https://www.stjude.org/CSTN/). We used 2 different antibodies for each histone mark.
- We used multiple cell lines and 8 xenografts to validate the ex-vivo data
- We assessed DNA damage and replicative stress by Comet assay, SKY analysis, gH2AX WB and IF, pRPA32 immunostaining and high throughput pharmacological assay
-We validated MYCN-induced metabolic reprograming using mitochondrial EM, Seahorse assay, lactate assay, Mitotracker green assay, TMRE assay GC analysis of tricarboxyclic acids metabolites and 14C-glucose and 14C-glutamine metabolomic trancing experiments.

However, the following experiments were done once:
Figure 1F-1H: this was a unique case in which there was discrepancy between COG FISH report and Next Generation sequencing report. This sample was carefully studied as described in the results section.
Figure 3B: This is the first and only O-PDX developed from ATRX-mutant neuroblastoma, as explained in the text.
Figure 5H: This experiment was done to confirm the high throughput screening and was done in a dose response.
Figure 5I: This experiment was done once. However, the ability of RA to reduce MYCN level is well-established in literature87.
Figure S1b-S1E: was done to all the clinical samples as mentioned in the material and methods
Figure S4H. This experiment was done once. However, this experiment is another evidence for increase in replicative stress when ATRX is inactivated and MYCN is ectopically expressed in different cells as shown in figure 5.
Figure S7C: This experiment was done once as another readout for increase ROS shown in figure S7D

Randomization

Human tumor samples were randomly selected from the COG Neuroblastoma biology bank to match the age and stage of disease criteria.

In orthotopic cell line mouse study, we enrolled the mice to receive doxycycline or stay on regular water randomly using a priori-made randomization table.
In genetic mouse models, mice were genotyped in the first 2 weeks after birth. Mice with the desirable genotypes were randomly enrolled at the time of weaning and followed up after.

Blinding

whenever possible, investigator were blinded to the different study groups or a standard protocols were applicable to all samples:

- Patients samples: all samples were subject to the same procedures including statistical analysis, Custom Capture and illumina sequencing, whole exome sequencing, MYCN FISH, ATRX IHC.
- Histopathologic review of Genetically modified animals was done by a veterinarian pathologist who was blind to the genotypes of the samples
- Genetic mouse models survival curves: all the mice were assessed in the same way for the presence and follow up of tumors. the outcome of survival or death of mice of tumors does not require a blinded investigators however all of the mouse morbidity events were reported first by one of animal care technicians who were completely blind to the study design.
CUT&RUN, ChIP Seq, WGBS,RNA, ChromHMM: were analyzed using standard bioinformatic protocols and QC that were applicable equally on all samples
Immunoblotting: standard protocols were equally applicable on all samples with positive and negative controls were included
Telomere FISH: was assessed blindly
Telomere QPCR: standard protocol was equally applicable on all samples with positive and negative controls were included
Cell Cycle Analysis: was assessed blindly with standard protocols were equally applicable on all samples
Colony Assays: assessed blindly, standard protocols were equally applicable on all samples
CRISPR/Cas9 Targeting ATRX and DAXX: standard protocols were equally applicable on all samples
Comet assay: assessed blindly
Spectral karyotyping (SKY) analysis : assessed blindly, standard protocols were equally applicable on all samples
Live Imaging: assessed blindly
In vivo Mixing Experiment: assessed blindly
Lactate Measurements: standard protocols were equally applicable on all samples
GC-MS-mediated analysis of 13C-labelled TCA metabolites: assessed blindly and standard protocols were equally applicable on all samples
Capillary electrophoresis- time of flight mass spectrometry (CE-TOFMS) for detecting metabolites: assessed blindly and standard protocols were equally applicable on all samples
pH Measurements: standard protocols were equally applicable on all samples
Mitotracker Green and TMRE Assays: assessed blindly and standard protocol were equally applicable on all samples including predetermined gating
Seahorse Assay: standard protocols were equally applicable on all samples
TEM Analysis of Mitochondria: assessed and scored blindly
Gamma H2AX Immunostaining: assessed blindly, standard protocols were equally applicable on all samples
ROS and glutathione levels: standard protocols were equally applicable on all samples
Drug screening:assessed blindly

Reporting for specific materials, systems and methods

We require information from authors about some types of materials, experimental systems and methods used in many studies. Here, indicate whether each material, system or method listed is relevant to your study. If you are not sure if a list item applies to your research, read the appropriate section before selecting a response.

Materials & experimental systems

n/a	Involved in the study
	☒ Antibodies
	☒ Eukaryotic cell lines
☒	Palaeontology
	☒ Animals and other organisms
	☒ Human research participants
☒	Clinical data

Methods

n/a	Involved in the study
	☒ ChIP-seq
	☒ Flow cytometry
☒	MRI-based neuroimaging

Antibodies

Antibodies used

rabbit anti-ATRX (1:1000, Abcam, ab97508), Lot# GR2672279
mouse anti-MYCN (1:1000, Santa cruz Biotechnology, sc-53993), B8-4B Lot# C1315
rabbit anti-GAPDH (1: 2,500, Abcam ab-9485), Lot# GR192135-3
rabbit anti-DAXX (M-112 1:1000, Santa cruz Biotechnology, sc-7152) Lot# D0715
mouse anti-b-actin (1: 5,000, Sigma, A1978). Lot# 065M4837V
mouse anti Phospho Ser139-histone H2AX antibody, Clone JBW301 (Millipore, Cat. # 5-636), 1:10,000 Lot#2884537
mouse ani-H3.3 antibodies Abnova, clone 2D7-H1 Cat# H00003021-M01)
rabbit anti-Phospho-RPA32 (S4/S8) (1:750, Bethyl, Cat# A300-245A)
rabbit anti- tyrosine hydroxylase diluted 1:500 (cat# AB152, EMD Millipore, Billerica, MA);
rabbit anti-synaptophysin diluted 1:400 (cat# ab7837; Abcam, Cambridge, UK);
rabbit anti-Ki-67 antigen (Clone SP6) diluted 1:50 (cat# RM-9106-S; Thermo Scientific, Fremont, CA).
rabbit anti-mouse IgG (Abcam, ab46540), secondary antibodies
donkey anti-mouse Cy3 IgG (Millipore, Cat. # AP192C), secondary antibodies
rabbit anti-mouse IgG (Abcam, ab46540), secondary antibodies
IRDye 800CW goat anti-mouse (1: 5,000, LiCore, 925-68070), secondary antibodies
IRDye 680CW goat anti-rabbit (1: 5,000, LiCore, 925-32211), secondary antibodies

Validation

- rabbit anti-ATRX (1:1000, Abcam, ab97508),One of the most commonly used antibody in the field. the manufacturer recommends this antibody for ICC/IF, IHC-P, IP, WB. Reactive against human and mouse. It has been used and referenced in at least 13 publications https://www.abcam.com/atrx-antibody-ab97508-references.html#top-948.
- mouse anti-MYCN (1:1000, Santa cruz Biotechnology, sc-53993). This antibody is recommended for WB, IP. Reactive against human, mouse and rat, it was used and referenced by at least 6 papers https://datasheets.scbt.com/sc-53993.pdf
- rabbit anti-GAPDH (1: 2,500, Abcam ab-9485). Validated for IHC-P, IP, ELISA, WB, IHC-Fr, ICC/IF, Flow Cyt by the manufacturer. reactive against: Mouse, Rat, Chicken, Dog, Human, Saccharomyces cerevisiae, Xenopus laevis, Schizosaccharomyces pombe, African green monkey. It has been used and referenced in over 1146 publications, https://www.abcam.com/gapdh-antibody-loading-control-ab9485-references.html#top-741
- rabbit anti-DAXX (M-112 1:1000, Santa cruz Biotechnology, sc-7152). the manufacturer recommended this antibody for WB, IP, IF, ELISA, reactive against: Human, mouse and rat. It has been used and referenced in over 8 publications http://datasheets.scbt.com/sc-7152.pdf
- mouse anti-b-actin (1: 5,000, Sigma, A1978). the manufacturer recommends this antibody for ARR, ICC, IF, IHC (f), WB. Reactive against pig, Hirudo medicinalis, bovine, rat, canine, feline, human, rabbit, carp, mouse, guinea pig, chicken, sheep. t has been used and referenced in over 2000 publications https://www.sigmaaldrich.com/catalog/search?interface=All&term=A1978&N=0&lang=en&region=US&focus=papers&mode=match+partialmax

mouse anti Phospho Ser139-histone H2AX antibody (Millipore, Cat. # 5-636). the manufacturer recommend this antibody for IHC, ChIP-Seq, WB, IF. reactive against human, mouse, rat. It has been used and referenced in over 200 publications. https://www.emdmillipore.com/US/en/product/Anti-phospho-Histone-H2A.X-Ser139-Antibody-clone-JBW301,MM_NF-05-636#documentation
- rabbit anti-Ki-67 antigen (Clone SP6) Thermo Scientific, this antibody was validaed by the manufacturer for IHC. reactive against human and other species (not tested). it was previously used and cited https://www.thermofisher.com/order/catalog/product/RM-9106-R7

-rabbit anti-synaptophysin abcam, ab7873. this antibody was validated by the manufacturer for IHC-FoFr, IHC-Fr, IHC-P, ICC/IF. reactive against human, mouse. this antibody was used and referenced in at least 8 publications. https://www.abcam.com/synaptophysin-antibody-ab7837-references.html#top-926

-Mouse ani-H3.3 antibodies Abnova, clone 2D7-H1, it was validated by the manufacturer for WB, IF, IHC, ELISA, reactive against human. Used in at least 8 publications. http://www.abnova.com/products/products_detail.asp?catalog_id=H00003021-M01

-Rabbit anti-Phospho-RPA32 (S4/S8). the manufacturer recommends it for WB, IP, IHC, ICC-IF. reactive against human and mouse. it has been used and referenced in at least 199 publications https://www.citeab.com/antibodies/654690-a300-245a-phospho-rpa32-s4-s8-antibody/publications

- rabbit anti- tyrosine hydroxylase. the manufacturer recommends this antibodies for ELISA, IF, IH, IH(P), IP and WB. reactive against human, mouse, rat, ferret. it was used and referenced in at least 100 publications. https://www.emdmillipore.com/US/en/product/Anti-Tyrosine-Hydroxylase-Antibody,MM_NF-AB152#documentation

-

Eukaryotic cell lines

Policy information about cell lines

Cell line source(s)	We provided this information in the Supplementary Table 2<br>CHLA90, LAN6: Children Oncology group<br>IMR-32, SKNBE2, SKNFI, G-292, WI-38 VA-132RA, SAOS2, U2OS, 293T, HeLa, SJSA1: ATCC<br>NB5: St Jude Children's Research Hospital<br>NBL-S: a valuable gift from Brodeur lab, Children's Hospital of Philadelphia (CHOP), Philadelphia, PA<br>SKNMM: a valuable gift from Cheung lab, Memorial Sloan Kettering Hospital, New York, NY<br>U251-ATRX, U251-EV, MOG-G-UVW-ATRX, MOG-G-UVW-EV: valuable gifts from Meeker/Heaphy lab, Johns Hopkins School of Medicine, Baltimore, MD
Authentication	We used STR microsatellite profiling to authenticate all cell lines used in this study (Supplementary Table 2)
Mycoplasma contamination	Mycoplasma testing was done on all the cells lines in this study and were negative for mycoplasma using Universal Mycoplasma Detection Kit (ATCC® 30-1012K™)
Commonly misidentified lines<br>(See ICLAC register)	We did not use any of the commonly misidentified cell lines

Animals and other organisms

Policy information about studies involving animals; ARRIVE guidelines recommended for reporting animal research

Laboratory animals	Patient-derived xenograft cells were maintained orthotopically in athymic nude (Jackson Laboratories, strain code 007850), NSG (Jackson Laboratories, strain code 005557), females 6-8 weeks old. or C57BL/6 scid mice (Jackson Laboratories, strain code 001913), females, age 6-8 weeks<br>In SKNMM-MYCN/ SKNMM-Cont orthotopic mixing experiment, we used NOD.CB17-Prkdcscid/J female mice 6-8 weeks old (Jackson laboratories, Stock # 001303).<br>Genetic mouse models for neuroanatomy were all C57BL/6<br>the number of the mice used from every strain is stated in the results and the supplemental Materials and Methods.
Wild animals	The study did not involve wild animals
Field-collected samples	The study did not involve samples collected from the field
Ethics oversight	Mouse studies were performed in a strict accordance with the recommendations in the Guide to Care and Use of Laboratory Animals of the National Institutes of Health. The protocol was approved by the Institutional Animal Care and Use Committee (IACUC) at St. Jude Children's Research Hospital. Protocol number 393

Note that full information on the approval of the study protocol must also be provided in the manuscript.

Human research participants

Policy information about studies involving human research participants

Population characteristics	Patients were eligible for inclusion in the analytic cohort if they enrolled in the COG<br>Neuroblastoma Biology study ANBL00B1 before treatment; had a confirmed diagnosis of<br>neuroblastoma; and had reported outcome data. Informed consent of the patients and/or<br>parents/legal guardians was obtained at the time of enrollment to ANBL00B1.<br>The demographic characteristics as well age covariants tested of the selected samples are listed in table1:<br>INSS stage: non-stage= 4 312 (65.8%), stage 4= 162 (34.2%)<br>Risk Group: low/intermediate = 375 (79.6%), high= 96 (20.4%)<br>Sex: female= 253 (53.4%), male= 221 (46.6%)<br>MYCN (FISH): not amplified= 431 (91.7%), amplified= 39 (8.3%)<br>MYCN (NGS): not amplified= 447 (94.1%), amplified= 28 (5.9%)<br>ALK: no mutation= 454 (95.6%), mutation= 21 (4.4%)<br>Ploidy: hyperdiploid= 277 (60.7%), diploid= 179 (39.3%)<br>11q LOH: no= 301 (91.8%), yes= 27 (8.2%)<br>1p LOH: no= 290 (88.4%), yes= 38 (11.6%)<br>Histology: favorable= 314 (69.8%), unfavorable= 136 (30.2%)<br>Age at Diagnosis: <18 months= 213 (44.9%), 18 mo-5 yrs= 76 (16.0%), 5–12 yrs= 152 (32.1%), >12 years= 33 (7.0%)<br>Grade: differentiating= 43 (13.2%), poorly/undifferentiated= 283 (86.8%)<br>MKI= low/intermediate= 298 (90.3%), high= 32 (9.7%)<br>Race: black= 55 (12.7%), other= 378 (87.3%)
Recruitment	Patients were eligible for inclusion in the analytic cohort if they enrolled in the COG<br>Neuroblastoma Biology study ANBL00B1 before treatment; had a confirmed diagnosis of

	neuroblastoma; and had reported outcome data. The selection of patients was random and we believe that there was no bias in data selection.
Ethics oversight	Institutional IRB at St. Jude Children's Research Hospital and the NCI Pediatric Cancer IRB

Note that full information on the approval of the study protocol must also be provided in the manuscript.

ChIP-seq

Data deposition

- [x] Confirm that both raw and final processed data have been deposited in a public database such as GEO.
- [x] Confirm that you have deposited or provided access to graph files (e.g. BED files) for the called peaks.

Data access links
May remain private before publication.

https://pecan.stjude.org/proteinpaint/study/mycn_nbl_2018

Files in database submission

BRD4-AB1-SJNBL043776_C1.bam
CTCF-AB1-SJNBL016371_C8.bam
H3K27Ac-AB1-SJNBL016371_C3.bam
H3K27Ac-AB2-SJNBL016371_C3.bam
H3K27me3-AB3-SJNBL016371_C3.bam
H3K36me3-AB2-SJNBL016371_C3.bam
H3K4me1-AB2-SJNBL016371_C3.bam
H3K4me2-AB1-SJNBL016371_C3.bam
H3K4me2-AB2-SJNBL016371_C3.bam
H3K4me3-AB2-SJNBL016371_C1.bam
H3K9-14Ac-AB2-SJNBL016371_C3.bam
H3K9-14Ac-AB3-SJNBL016371_C2.bam
H3K9-14Ac-AB3-SJNBL016371_C3.bam
H3K9me3-AB1-SJNBL016371_C3.bam
H3K9me3-AB1-SJNBL043776_C1.bam
H3K9me3-AB2-SJNBL016371_C3.bam
INPUT-SJNBL016371_C3.bam
RNAPolII-AB1-SJNBL043138_C1.bam
BRD4-AB1-SJNBL043776_C2.bam
CTCF-AB1-SJNBL040760_C9.bam
H3K27Ac-AB1-SJNBL040760_C3.bam
H3K27Ac-AB2-SJNBL040760_C3.bam
H3K27me3-AB3-SJNBL040760_C3.bam
H3K36me3-AB1-SJNBL040760_C3.bam
H3K36me3-AB2-SJNBL040760_C3.bam
H3K4me1-AB2-SJNBL040760_C3.bam
H3K4me2-AB1-SJNBL040760_C3.bam
H3K4me2-AB2-SJNBL040760_C2.bam
H3K4me3-AB2-SJNBL040760_C1.bam
H3K9-14Ac-AB2-SJNBL040760_C3.bam
H3K9-14Ac-AB3-SJNBL040760_C1.bam
H3K9-14Ac-AB3-SJNBL040760_C3.bam
H3K9me3-AB1-SJNBL040760_C3.bam
H3K9me3-AB1-SJNBL043776_C2.bam
H3K9me3-AB2-SJNBL040760_C3.bam
INPUT-SJNBL040760_C5.bam
RNAPolII-AB1-SJNBL043138_C2.bam
BRD4-AB1-SJNBL043776_C3.bam
CTCF-AB1-SJNBL016370_C10.bam
H3K27Ac-AB1-SJNBL016370_C4.bam
H3K27Ac-AB2-SJNBL016370_C4.bam
H3K27me3-AB3-SJNBL016370_C4.bam
H3K36me3-AB1-SJNBL016370_C4.bam
H3K36me3-AB2-SJNBL016370_C4.bam
H3K4me1-AB2-SJNBL016370_C2.bam
H3K4me1-AB2-SJNBL016370_C4.bam
H3K4me2-AB1-SJNBL016370_C4.bam
H3K4me2-AB2-SJNBL016370_C3.bam
H3K4me3-AB2-SJNBL016370_C1.bam
H3K9-14Ac-AB2-SJNBL016370_C4.bam
H3K9-14Ac-AB3-SJNBL016370_C2.bam

H3K9-14Ac-AB3-SJNBL016370_C4.bam
H3K9me3-AB1-SJNBL016370_C4.bam
H3K9me3-AB1-SJNBL043776_C3.bam
H3K9me3-AB2-SJNBL016370_C4.bam
INPUT-SJNBL016370_C5.bam
RNAPolII-AB1-SJNBL043138_C3.bam
BRD4-AB1-SJNBL013762_X3.bam
CTCF-AB1-SJNBL013762_X4.bam
CTCF-AB1-SJNBL013762_X5.bam
H3K27Ac-AB2-SJNBL013762_X4.bam
H3K27Ac-AB2-SJNBL013762_X5.bam
H3K27me3-AB3-SJNBL013762_X2.bam
H3K27me3-AB3-SJNBL013762_X4.bam
H3K36me3-AB1-SJNBL013762_X4.bam
H3K36me3-AB1-SJNBL013762_X5.bam
H3K36me3-AB2-SJNBL013762_X4.bam
H3K36me3-AB2-SJNBL013762_X5.bam
H3K4me1-AB2-SJNBL013762_X4.bam
H3K4me1-AB2-SJNBL013762_X6.bam
H3K4me2-AB1-SJNBL013762_X5.bam
H3K4me2-AB2-SJNBL013762_X4.bam
H3K4me2-AB2-SJNBL013762_X5.bam
H3K4me3-AB2-SJNBL013762_X2.bam
H3K4me3-AB2-SJNBL013762_X4.bam
H3K9-14Ac-AB3-SJNBL013762_X2.bam
H3K9-14Ac-AB3-SJNBL013762_X4.bam
H3K9me3-AB1-SJNBL013762_X4.bam
H3K9me3-AB2-SJNBL013762_X4.bam
H3K9me3-AB2-SJNBL013762_X5.bam
INPUT-SJNBL013762_X5.bam
RNAPolII-AB1-SJNBL013762_X3.bam
BRD4-AB1-SJNBL006108_X1.bam
CTCF-AB1-SJNBL006108_X2.bam
CTCF-AB1-SJNBL006108_X3.bam
H3K27Ac-AB2-SJNBL006108_X2.bam
H3K27Ac-AB2-SJNBL006108_X3.bam
H3K27me3-AB1-SJNBL006108_X2.bam
H3K27me3-AB1-SJNBL006108_X3.bam
H3K36me3-AB1-SJNBL006108_X2.bam
H3K36me3-AB1-SJNBL006108_X3.bam
H3K36me3-AB2-SJNBL006108_X2.bam
H3K36me3-AB2-SJNBL006108_X3.bam
H3K4me1-AB2-SJNBL006108_X2.bam
H3K4me1-AB2-SJNBL006108_X3.bam
H3K4me2-AB2-SJNBL006108_X2.bam
H3K4me2-AB2-SJNBL006108_X3.bam
H3K4me3-AB2-SJNBL006108_X2.bam
H3K4me3-AB2-SJNBL006108_X3.bam
H3K9-14Ac-AB3-SJNBL006108_X2.bam
H3K9-14Ac-AB3-SJNBL006108_X3.bam
H3K9me3-AB1-SJNBL006108_X2.bam
H3K9me3-AB2-SJNBL006108_X2.bam
H3K9me3-AB2-SJNBL006108_X3.bam
INPUT-SJNBL006108_X2.bam
RNAPolII-AB1-SJNBL006108_X1.bam
BRD4-AB1-SJNBL013763_X2.bam
CTCF-AB1-SJNBL013763_X3.bam
CTCF-AB1-SJNBL013763_X4.bam
H3K27Ac-AB2-SJNBL013763_X3.bam
H3K27Ac-AB2-SJNBL013763_X4.bam
H3K27me3-AB1-SJNBL013763_X7.bam
H3K36me3-AB2-SJNBL013763_X3.bam
H3K36me3-AB2-SJNBL013763_X4.bam
H3K4me1-AB2-SJNBL013763_X3.bam
H3K4me1-AB2-SJNBL013763_X4.bam
H3K4me2-AB2-SJNBL013763_X3.bam
H3K4me2-AB2-SJNBL013763_X4.bam
H3K4me3-AB2-SJNBL013763_X3.bam

H3K4me3-AB2-SJNBL013763_X4.bam
H3K9-14Ac-AB3-SJNBL013763_X3.bam
H3K9-14Ac-AB3-SJNBL013763_X4.bam
H3K9me3-AB2-SJNBL013763_X3.bam
H3K9me3-AB2-SJNBL013763_X4.bam
INPUT-SJNBL013763_X4.bam
RNAPolII-AB1-SJNBL013763_X2.bam
BRD4-AB1-SJNBL030014_D2.bam
CTCF-AB1-SJNBL030014_D2.bam
H3K27Ac-AB1-SJNBL030014_D2.bam
H3K27me3-AB1-SJNBL030014_D2.bam
H3K36me3-AB1-SJNBL030014_D2.bam
H3K4me1-AB1-SJNBL030014_D2.bam
H3K4me2-AB1-SJNBL030014_D2.bam
H3K4me3-AB1-SJNBL030014_D2.bam
H3K9-14Ac-AB1-SJNBL030014_D2.bam
H3K9me3-AB1-SJNBL030014_D2.bam
INPUT-SJNBL030014_D2.bam
RNAPolII-AB1-SJNBL030014_D2.bam
BRD4-AB1-SJNBL015724_X2.bam
CTCF-AB1-SJNBL015724_X4.bam
CTCF-AB1-SJNBL015724_X5.bam
H3K27Ac-AB2-SJNBL015724_X4.bam
H3K27Ac-AB2-SJNBL015724_X5.bam
H3K27me3-AB1-SJNBL015724_X6.bam
H3K36me3-AB2-SJNBL015724_X4.bam
H3K36me3-AB2-SJNBL015724_X5.bam
H3K4me1-AB2-SJNBL015724_X4.bam
H3K4me1-AB2-SJNBL015724_X5.bam
H3K4me1-AB2-SJNBL015724_X7.bam
H3K4me2-AB2-SJNBL015724_X4.bam
H3K4me2-AB2-SJNBL015724_X5.bam
H3K4me3-AB2-SJNBL015724_X4.bam
H3K4me3-AB2-SJNBL015724_X5.bam
H3K9-14Ac-AB3-SJNBL015724_X4.bam
H3K9-14Ac-AB3-SJNBL015724_X5.bam
H3K9me3-AB2-SJNBL015724_X4.bam
H3K9me3-AB2-SJNBL015724_X5.bam
INPUT-SJNBL015724_X7.bam
RNAPolII-AB1-SJNBL015724_X3.bam
BRD4-AB1-SJNBL047443_X2.bam
BRD4-AB1-SJNBL047443_X3.bam
CTCF-AB1-SJNBL047443_X6.bam
CTCF-AB1-SJNBL047443_X7.bam
H3K27Ac-AB2-SJNBL047443_X4.bam
H3K27Ac-AB2-SJNBL047443_X5.bam
H3K27me3-AB3-SJNBL047443_X4.bam
H3K27me3-AB3-SJNBL047443_X5.bam
H3K36me3-AB2-SJNBL047443_X4.bam
H3K36me3-AB2-SJNBL047443_X5.bam
H3K4me1-AB2-SJNBL047443_X4.bam
H3K4me1-AB2-SJNBL047443_X5.bam
H3K4me2-AB2-SJNBL047443_X4.bam
H3K4me2-AB2-SJNBL047443_X5.bam
H3K4me3-AB1-SJNBL047443_X2.bam
H3K4me3-AB1-SJNBL047443_X3.bam
H3K9-14Ac-AB1-SJNBL047443_X6.bam
H3K9-14Ac-AB3-SJNBL047443_X5.bam
H3K9me3-AB1-SJNBL047443_X2.bam
H3K9me3-AB1-SJNBL047443_X3.bam
H3K9me3-AB2-SJNBL047443_X4.bam
H3K9me3-AB2-SJNBL047443_X5.bam
INPUT-SJNBL047443_X5.bam
RNAPolII-AB1-SJNBL047443_X2.bam
RNAPolII-AB1-SJNBL047443_X3.bam
BRD4-AB1-SJNBL006105_X6.bam
BRD4-AB1-SJNBL006105_X8.bam
CTCF-AB1-SJNBL006105_X7.bam

CTCF-AB1-SJNBL006105_X8.bam
H3K27Ac-AB1-SJNBL006105_X3.bam
H3K27Ac-AB1-SJNBL006105_X4.bam
H3K27Ac-AB2-SJNBL006105_X10.bam
H3K27Ac-AB2-SJNBL006105_X9.bam
H3K27me3-AB1-SJNBL006105_X3.bam
H3K27me3-AB1-SJNBL006105_X4.bam
H3K27me3-AB3-SJNBL006105_X1.bam
H3K27me3-AB3-SJNBL006105_X2.bam
H3K36me3-AB1-SJNBL006105_X3.bam
H3K36me3-AB1-SJNBL006105_X4.bam
H3K36me3-AB2-SJNBL006105_X10.bam
H3K36me3-AB2-SJNBL006105_X9.bam
H3K4me1-AB1-SJNBL006105_X3.bam
H3K4me1-AB1-SJNBL006105_X4.bam
H3K4me1-AB2-SJNBL006105_X1.bam
H3K4me1-AB2-SJNBL006105_X2.bam
H3K4me2-AB1-SJNBL006105_X3.bam
H3K4me2-AB1-SJNBL006105_X4.bam
H3K4me2-AB2-SJNBL006105_X10.bam
H3K4me2-AB2-SJNBL006105_X9.bam
H3K4me3-AB1-SJNBL006105_X3.bam
H3K4me3-AB1-SJNBL006105_X4.bam
H3K4me3-AB2-SJNBL006105_X1.bam
H3K4me3-AB2-SJNBL006105_X2.bam
H3K9-14Ac-AB3-SJNBL006105_X1.bam
H3K9-14Ac-AB3-SJNBL006105_X2.bam
H3K9me3-AB1-SJNBL006105_X7.bam
H3K9me3-AB1-SJNBL006105_X8.bam
H3K9me3-AB2-SJNBL006105_X9.bam
INPUT-SJNBL006105_X10.bam
RNAPolII-AB1-SJNBL006105_X6.bam
RNAPolII-AB1-SJNBL006105_X8.bam
BRD4-AB1-SJNBL013761_X2.bam
CTCF-AB1-SJNBL013761_X3.bam
CTCF-AB1-SJNBL013761_X4.bam
H3K27Ac-AB2-SJNBL013761_X3.bam
H3K27Ac-AB2-SJNBL013761_X4.bam
H3K27me3-AB1-SJNBL013761_X6.bam
H3K27me3-AB3-SJNBL013761_X3.bam
H3K36me3-AB2-SJNBL013761_X3.bam
H3K36me3-AB2-SJNBL013761_X4.bam
H3K4me1-AB2-SJNBL013761_X3.bam
H3K4me1-AB2-SJNBL013761_X4.bam
H3K4me2-AB2-SJNBL013761_X3.bam
H3K4me2-AB2-SJNBL013761_X4.bam
H3K4me3-AB2-SJNBL013761_X3.bam
H3K4me3-AB2-SJNBL013761_X4.bam
H3K9-14Ac-AB3-SJNBL013761_X3.bam
H3K9-14Ac-AB3-SJNBL013761_X4.bam
H3K9me3-AB2-SJNBL013761_X3.bam
H3K9me3-AB2-SJNBL013761_X4.bam
INPUT-SJNBL013761_X3.bam
RNAPolII-AB1-SJNBL013761_X2.bam
BRD4-AB1-SJNBL012407_X2.bam
CTCF-AB1-SJNBL012407_X3.bam
CTCF-AB1-SJNBL012407_X5.bam
H3K27Ac-AB2-SJNBL012407_X3.bam
H3K27Ac-AB2-SJNBL012407_X4.bam
H3K27me3-AB3-SJNBL012407_X3.bam
H3K27me3-AB3-SJNBL012407_X4.bam
H3K36me3-AB1-SJNBL012407_X3.bam
H3K36me3-AB1-SJNBL012407_X4.bam
H3K36me3-AB2-SJNBL012407_X3.bam
H3K36me3-AB2-SJNBL012407_X4.bam
H3K4me1-AB2-SJNBL012407_X3.bam
H3K4me1-AB2-SJNBL012407_X4.bam
H3K4me2-AB1-SJNBL012407_X4.bam

H3K4me2-AB2-SJNBL012407_X3.bam
H3K4me2-AB2-SJNBL012407_X4.bam
H3K4me3-AB2-SJNBL012407_X3.bam
H3K4me3-AB2-SJNBL012407_X4.bam
H3K9-14Ac-AB3-SJNBL012407_X3.bam
H3K9-14Ac-AB3-SJNBL012407_X4.bam
H3K9me3-AB1-SJNBL012407_X3.bam
H3K9me3-AB1-SJNBL012407_X4.bam
H3K9me3-AB2-SJNBL012407_X3.bam
H3K9me3-AB2-SJNBL012407_X4.bam
INPUT-SJNBL012407_X4.bam
RNAPolII-AB1-SJNBL012407_X2.bam
BRD4-AB1-SJNBL043776_C4.bam
CTCF-AB1-SJNBL040762_C9.bam
H3K27Ac-AB1-SJNBL040762_C3.bam
H3K27Ac-AB2-SJNBL040762_C3.bam
H3K27me3-AB3-SJNBL040762_C8.bam
H3K36me3-AB2-SJNBL040762_C3.bam
H3K4me1-AB2-SJNBL040762_C3.bam
H3K4me2-AB1-SJNBL040762_C3.bam
H3K4me2-AB2-SJNBL040762_C2.bam
H3K4me3-AB2-SJNBL040762_C1.bam
H3K9-14Ac-AB2-SJNBL040762_C3.bam
H3K9-14Ac-AB3-SJNBL040762_C1.bam
H3K9-14Ac-AB3-SJNBL040762_C3.bam
H3K9me3-AB1-SJNBL040762_C3.bam
H3K9me3-AB1-SJNBL043776_C4.bam
H3K9me3-AB2-SJNBL040762_C3.bam
INPUT-SJNBL040762_C9.bam
RNAPolII-AB1-SJNBL043138_C4.bam
BRD4-AB1-SJNBL043776_C5.bam
CTCF-AB1-SJNBL044375_C9.bam
H3K27Ac-AB1-SJNBL044375_C4.bam
H3K27Ac-AB2-SJNBL044375_C4.bam
H3K27me3-AB3-SJNBL044375_C3.bam
H3K36me3-AB2-SJNBL044375_C4.bam
H3K4me1-AB2-SJNBL044375_C4.bam
H3K4me2-AB1-SJNBL044375_C4.bam
H3K4me2-AB2-SJNBL044375_C1.bam
H3K4me3-AB2-SJNBL044375_C4.bam
H3K9-14Ac-AB2-SJNBL044375_C4.bam
H3K9-14Ac-AB3-SJNBL044375_C4.bam
H3K9me3-AB1-SJNBL044375_C4.bam
H3K9me3-AB1-SJNBL043776_C5.bam
H3K9me3-AB2-SJNBL044375_C4.bam
INPUT-SJNBL044375_C4.bam
RNAPolII-AB1-SJNBL043138_C5.bam
BRD4-AB1-SJNBL043776_C6.bam
CTCF-AB1-SJNBL040761_C8.bam
H3K27Ac-AB1-SJNBL040761_C3.bam
H3K27Ac-AB2-SJNBL040761_C3.bam
H3K27me3-AB3-SJNBL040761_C8.bam
H3K36me3-AB2-SJNBL040761_C3.bam
H3K4me1-AB2-SJNBL040761_C3.bam
H3K4me2-AB1-SJNBL040761_C3.bam
H3K4me2-AB2-SJNBL040761_C2.bam
H3K4me3-AB2-SJNBL040761_C1.bam
H3K9-14Ac-AB2-SJNBL040761_C3.bam
H3K9-14Ac-AB3-SJNBL040761_C1.bam
H3K9-14Ac-AB3-SJNBL040761_C3.bam
H3K9me3-AB1-SJNBL040761_C3.bam
H3K9me3-AB1-SJNBL043776_C6.bam
H3K9me3-AB2-SJNBL040761_C3.bam
INPUT-SJNBL040761_C5.bam
RNAPolII-AB1-SJNBL043138_C6.bam
BRD4-AB1-SJNBL043776_C7.bam
CTCF-AB1-SJNBL010856_C13.bam
H3K27Ac-AB1-SJNBL010856_C6.bam

H3K27Ac-AB2-SJNBL010856_C6.bam
H3K27me3-AB3-SJNBL010856_C12.bam
H3K27me3-AB3-SJNBL010856_C6.bam
H3K36me3-AB1-SJNBL010856_C6.bam
H3K36me3-AB2-SJNBL010856_C6.bam
H3K4me1-AB2-SJNBL010856_C6.bam
H3K4me2-AB1-SJNBL010856_C6.bam
H3K4me2-AB2-SJNBL010856_C5.bam
H3K4me3-AB2-SJNBL010856_C4.bam
H3K9-14Ac-AB2-SJNBL010856_C6.bam
H3K9-14Ac-AB3-SJNBL010856_C4.bam
H3K9-14Ac-AB3-SJNBL010856_C6.bam
H3K9me3-AB1-SJNBL010856_C6.bam
H3K9me3-AB1-SJNBL043776_C7.bam
H3K9me3-AB2-SJNBL010856_C6.bam
INPUT-SJNBL010856_C13.bam
RNAPolII-AB1-SJNBL043138_C7.bam
BRD4-AB1-SJNBL043776_C8.bam
CTCF-AB1-SJNBL016372_C9.bam
H3K27Ac-AB1-SJNBL016372_C4.bam
H3K27Ac-AB2-SJNBL016372_C4.bam
H3K27me3-AB3-SJNBL016372_C2.bam
H3K27me3-AB3-SJNBL016372_C4.bam
H3K36me3-AB2-SJNBL016372_C4.bam
H3K4me1-AB2-SJNBL016372_C4.bam
H3K4me2-AB1-SJNBL016372_C4.bam
H3K4me2-AB2-SJNBL016372_C3.bam
H3K4me3-AB2-SJNBL016372_C1.bam
H3K9-14Ac-AB2-SJNBL016372_C4.bam
H3K9-14Ac-AB3-SJNBL016372_C2.bam
H3K9-14Ac-AB3-SJNBL016372_C4.bam
H3K9me3-AB1-SJNBL016372_C4.bam
H3K9me3-AB1-SJNBL043776_C8.bam
H3K9me3-AB2-SJNBL016372_C4.bam
INPUT-SJNBL016372_C4.bam

Genome browser session
(e.g. UCSC)

EMBL-EBI database, accession number EGAS00001003257

Methodology

Replicates

We used 8 different cells lines (3 MYCN amplified, 2 ATRX mutant, 1 low level MYCN amplification and 2 with no MYCN amplification or ATRX mutations)
For xenografts, we used 8 different patient derived xenografts (4 MYCN amplified, 3 with no MYCN amplification and the only available one with ATRX mutations)
For every histone mark, we used 2 different antibodies

Sequencing depth

Single reads of 50-bp
reads were at least 15 M reads

Antibodies

H3K4me3, Active Motif, 39159 Lot# 12613005
H3K4me3; Diagenode, C15410003-50, A-5051001P
H3K4me2; Active Motif, 39141, 01008001 H3K4me2; Abcam, Ab7766, GR160184-1 H3K4me1; Abcam, Ab8895, GR-24233-1
H3K4me1; Active Motif, 39297, 01714002 H3K9/14Ac; Life Tech, 49-1010, A3800004 H3K9/14Ac; Diagenode, C15410200, A1756D H3K27Ac; Abcam, ab4729, GR183922-2 H3K27Ac; Active Motif, 39133, 31614008 H3K36me3; Abcam, ab9050, GR204353-1
H3K36me3; Active Motif, 61101, 32412003
H3K27me3; Millipore, 07-449, 2662971 & 2607768
H3K27me3; Active Motif, 39155, 31814017 H3K9me3; Invitrogen, 491008, A1675001P H3K9me3; Active Motif, 39161, 13509002
CTCF; Active Motif, 61311, 34614003
N-MYC: Active Motif, 61185

-ChIP for N-MYC, BRD4, PolII and H3K9me3 was done by Active Motif but Sequenced and analyzed at St. Jude Research Hospital

Peak calling parameters	MACS2 (version 2.0.10.20131216) were used to call peaks for MYCN ChIP-Seq data with FDR corrected p-value 0.05(--nomodel –extsize FRAGMENTSIZE, where FRAGMENTSIZE was estimated by SPP)
Data quality	The quality control of the data followed ENCODE criteria. We calculated relative strand correlation value (RSC) and estimated the fragment size under support of R (version 2.14.0) with packages caTools (version 1.17) and bitops (version 1.0-6). We required at least 10M unique reads for point source factors H3K4me2/3, H3K9/14Ac, H3K27Ac, CTCF, RNAPolII, BRD4, MYCN) with RSC > 1, 20M unique reads for broad markers (H3K4me1, H3K9me3, H3K27me3, H3K36me3) and 10M unique mapped reads for INPUTs with RSC < 1. All samples were manually inspected and the SPP (version 1.1) was used to generate the cross-correlation plot. Then we generated bigwig files from the best fragment size (the smallest fragment size estimated by SPP). Bigwig files were examined using IGV genome browser for clear peaks and low background noise.
Software	ChIP-Seq reads were aligned to human genome hg19 (GRCh37-lite) using BWA software (version 0.7.12-r1039, default parameter) and the duplicated reads were marked using Picard software. We kept only nonduplicated reads for the analysis using samtools (parameter "-q 1 -F 1024" version 1.2).

Flow Cytometry

Plots

Confirm that:

- [x] The axis labels state the marker and fluorochrome used (e.g. CD4-FITC).
- [x] The axis scales are clearly visible. Include numbers along axes only for bottom left plot of group (a 'group' is an analysis of identical markers).
- [x] All plots are contour plots with outliers or pseudocolor plots.
- [x] A numerical value for number of cells or percentage (with statistics) is provided.

Methodology

Sample preparation	For Cell cycle analysis, cells were trypsinized and harvested, pelleted and suspended in 1-ml propidium iodide solution (0.05 mg/ml propidium iodide (Sigma, Cat. # P4864), 0.1% sodium citrate, 0.1% Triton X-100). The cells were then treated with 10-ml 0.2 mg/ml of ribonuclease A (Colbiochem, Cat. # 556746) for 30 minutes at room temperature, filtered through 40-um nylon mesh and analyzed by flow cytometry for the DNA content.<br>MitoTracker™ Green FM (Thermofisher # M7514) or Tetramethylrhodamine ethyl ester perchlorate (TMRE, Thermofisher # T669) was added directly to the medium to a final concentration of 200 nM or 50 nM respectively. After 20 minutes incubation at 37C, the medium was removed and the cells were washed twice in 1X PBS and harvested. Fluorescent intensity was measured in at least 20,000 cells as suggested by the manufacturer. Data are presented as a geomean 95% confidence interval. The comparison between the fluorescence intensity in cells with or without doxycycline was done using the two-tailed T-Test.<br>For CellRox Green, cells grow in 10-cm plates. CellRox Green was added to the medium 1:500 and incubated for 30 minutes then cells were harvested and analyzed using flowcytometry.
Instrument	BD FACSAria Fusion
Software	Diva 8.01
Cell population abundance	not relevant, no cells sorted
Gating strategy	A Primary gate was established on FSC/Log-SSC. A second gate was then drawn using SSC-W/FSC to identify Doublets.

- [x] Tick this box to confirm that a figure exemplifying the gating strategy is provided in the Supplementary Information.
